# Essential Oils as a Source of Anticancer Molecules: Critical Assessment of Current Evidence and Methodological Limitations—A Systematic Review

**DOI:** 10.3390/ijms27104379

**Published:** 2026-05-14

**Authors:** Renato Spigarelli, Maria Chiara Valerii, Alberto Bernacchi, Nikolas Kostantine Dussias, Lina Mbirki, Enzo Spisni

**Affiliations:** 1 Department of Biological, Geological, and Environmental Sciences (BiGeA), University of Bologna, Via Selmi 3, 40126 Bologna, Italy; mariachiara.valerii2@unibo.it (M.C.V.); enzo.spisni@unibo.it (E.S.); 2 Inflammatory Bowel Disease (IBD) Unit, Istituti di Ricovero e Cura a Carattere Scientifico (IRCCS), Azienda Ospedaliero-Universitaria di Bologna, University of Bologna, Via Massarenti 9, 40138 Bologna, Italy; nikolas.dussias@studio.unibo.it; 3 Department of Medical and Surgical Sciences, University of Bologna, Via Massarenti 9, 40138 Bologna, Italy; 4 Laboratory of Integrated Physiology (LR17ES02), Faculty of Sciences of Bizerte, University of Carthage, Bizerte 7021, Tunisia; linambirki@gmail.com

**Keywords:** essential oils, anticancer activity, natural products, tumor selectivity, experimental methodology

## Abstract

Essential oils (EOs) and their bioactive constituents have attracted growing interest as potential anticancer agents because they can target multiple pathways involved in tumor progression. However, the literature on their anticancer activity is highly heterogeneous and often limited by methodological weaknesses that reduce the reliability and translational value of the reported findings. This systematic review critically assessed the anticancer activity of EOs and EO-derived compounds by considering only studies that met defined methodological criteria. A PubMed search identified 872 articles published between 2015 and 2026, of which 97 were retained after screening based on EO chemical characterization, evaluation of cancer selectivity using non-tumoral control cells, and direct assessment of EO-driven anticancer effects. Across different tumor models, EOs and their constituents consistently showed selective cytotoxic or antiproliferative activity, commonly associated with oxidative stress, mitochondrial dysfunction, apoptosis, cell-cycle arrest, and modulation of oncogenic pathways. Some studies also reported reduced migration, invasion, and tumor-promoting signaling, while nanoformulation improved stability and efficacy in selected models. Overall, despite encouraging preclinical evidence, the translational potential of EO-based anticancer strategies remains limited by recurrent methodological shortcomings and insufficient in vivo validation. Standardized experimental criteria will be essential to improve reproducibility and support future clinical development.

## 1. Introduction

The term ‘essential oil’ originated in the 16th century and was derived from the concept of ‘Quinta essentia’ introduced by Paracelsus (1493–1541), a pioneer in this field, developing plant extracts and herbal medicines. He believed that the distillation process extracted the most important part of the plant, separating the ‘essential’ components from the ‘non-essential’ [1].

Nowadays, the definition of essential oils (EOs) has been established by the International Organization for Standardization (ISO), which defines them as “products obtained from a natural raw material of plant origin, by steam distillation, by mechanical processes from the epicarp of citrus fruits or by dry distillation, after separation of the aqueous phase–if any–by physical processes” and specifies that “the essential oil can undergo physical treatments, which do not result in any significant change in its composition (e.g., filtration, decantation, centrifugation)” [2].

Chemically, EOs are complex mixtures of various lipophilic volatile compounds that can be extracted from all parts of aromatic plants, such as rhizomes, leaves, fruits and seeds [3]. The constituents of EOs belong to different classes of low molecular weight organic compounds and most of them are known for their wide-ranging biological activity [4]. Based on their chemical structure, the individual components of EOs can be classified into four main groups: terpenes, terpenoids, phenylpropanoids, and ‘others’ (a group indicating the remaining compounds, which are usually present in minor percentages) [5]. Terpenes and their derivatives terpenoids represent the major classes of chemical components found in EOs, with over 50,000 molecules so far identified [6,7]. Terpenes are composed of five-carbon isoprene units (2-methylbuta-1,3-diene), which can be combined in numerous ways (from one to thousands of units) to form a wide variety of structures, chemically and functionally different [8,9].

Terpenoids are a modified class of terpenes that contain oxygen molecules, formed via biochemical modifications of terpenes, such as the addition or removal of methyl groups. They can be classified into several subgroups, including alcohols, ketones, aldehydes, esters, phenols, ethers, and epoxides [5,10].

Phenylpropanoids are compounds characterized by a six-carbon aromatic phenyl group attached to a three-carbon side chain. This side chain, originating from cinnamic acid or its derivatives, can be either saturated or unsaturated and may feature a diverse array of functional groups or structural modifications. These modifications can include esterification with sugars or organic acids, the presence of carboxylic acids and hydroxyl groups, modifications to the carbonyl function, and other structural variations, all contributing to the extensive chemical diversity of phenylpropanoids produced in plant biosynthesis [11,12,13].

The “other” group also includes various degradation products that can originate from unsaturated fatty acids, terpenes, glycosides, sulphur-containing compounds, nitrogen-containing compounds and amino acids; some of these compounds may also be end products of degradation themselves [5,10].

EOs have been used by humans since ancient times and are still employed today for medicinal purposes in traditional medicine, which has now developed a refined knowledge about their therapeutic properties, as extensively documented by the scientific literature. Their main therapeutic activities include anti-inflammatory, antioxidant, antifungal, antibacterial, antiviral, immunomodulatory, antiparasitic, sedative, spasmolytic, antiseptic, and anticancer [14,15,16,17]. In particular, the anti-tumor effects of EOs have gained increasing interest given their strong and often selective activity on tumor cells. Considering the therapeutic barriers posed by drug resistance and adverse effects of standard anticancer therapeutic protocols, EOs and their bioactive components have emerged as novel potential therapeutic options [18]. Not only EOs but also their individual molecules have demonstrated anticancer properties by inhibiting transformed cell proliferation, inducing apoptosis, modulating the tumor microenvironment and offering a complementary approach to conventional treatments, to reduce tumor growth and mitigate cancer-related risk factors [18,19]. Despite the promising results obtained in vitro and in animal models, the anticancer activity of EOs in humans remains largely unexplored. This gap is attributable not only to the intrinsic complexity of these mixtures, but also to the limited reproducibility and methodological weakness of many studies, highlighting the need to clearly define universally adopted criteria to substantiate experimental evidence of the anticancer activity of EOs.

For this reason, we reviewed and selected reliable studies on EOs, focusing on their anticancer effects, including their individual constituents. The objectives of this systematic review are to highlight the main limitations and emerging challenges in defining the anticancer properties of EOs.

## 2. Methods

This systematic review was conducted in accordance with the PRISMA (Preferred Reporting Items for Systematic Reviews and Meta-Analyses) guidelines (see PRISMA 2020 Checklist in the Supplementary Materials). No prior protocol registration (e.g., PROSPERO) was performed for this study. Due to the heterogeneity of the included preclinical studies, no formal risk-of-bias assessment tool was applied.

The PubMed database was queried using the “advanced” search tool with the following string:

(“essential oil*”[Title/Abstract] AND (“anticancer*”[Title/Abstract] OR “antitumoral*”[Title/Abstract])) NOT (“review”[Publication Type]) AND 2015:2026[DP].

In addition, Web of Science (WoS) and Scopus databases were searched using the following queries: WoS: “TS = (“essential oil*” AND (anticancer* OR antitumor*)), NOT TS = (review), Timespan: 2015–2026”. Scopus: “TITLE-ABS-KEY (“essential oil*” AND (anticancer* OR antitumor*)), AND PUBYEAR > 2014 AND PUBYEAR < 2027, AND NOT DOCTYPE (re)”.

The time range was adjusted to include studies published up to 31 January 2026.

The retrieved literature was scanned and filtered based on the following inclusion criteria: the article must have been written in English; the EO must have been characterized or the tested compound must be a well-established constituent of an EO; the study must have been conducted using EOs or their components as the primary anticancer treatment, either in vivo, or in vitro with at least one normal, non-cancerous cell line of the same species as a control for selective cytotoxicity against malignant cells.

Since “in vitro studies with non-cancerous cells isolated from the same tissue as the cancerous cells” was considered overly restrictive, all types of selective cytotoxicity controls were taken into account for this review, including studies in which selective, concentration-dependent anticancer effects were demonstrated without formal IC_50_ determination, as well as studies providing direct EO-driven anticancer activity supported by mechanistic evidence.

Accordingly, studies evaluating combinations of EOs (or their individual constituents) with standard anticancer drugs were included in the results section only when EO-alone data fulfilling these criteria were reported; otherwise, such studies were considered exclusively in the discussion.

For in vivo studies, only those assessing a direct antitumor effect (e.g., tumor growth inhibition, tumor burden reduction, survival benefit, or chemopreventive efficacy) were considered.

No automation tool was used in the screening of the articles.

To ensure the objectivity of the selection process, the rigor of the filtering process and the final eligibility of the selected articles, all authors of this manuscript screened the title and abstract of each paper. Subsequently, the full texts were carefully read independently by two authors (A.B. and R.S.). Only studies that reached consensus from both independent reviewers were retained and included in this review. This double-check method ensured minimal bias and reduced the arbitrariness of the selection process.

## 3. Results

**Figure 1:**
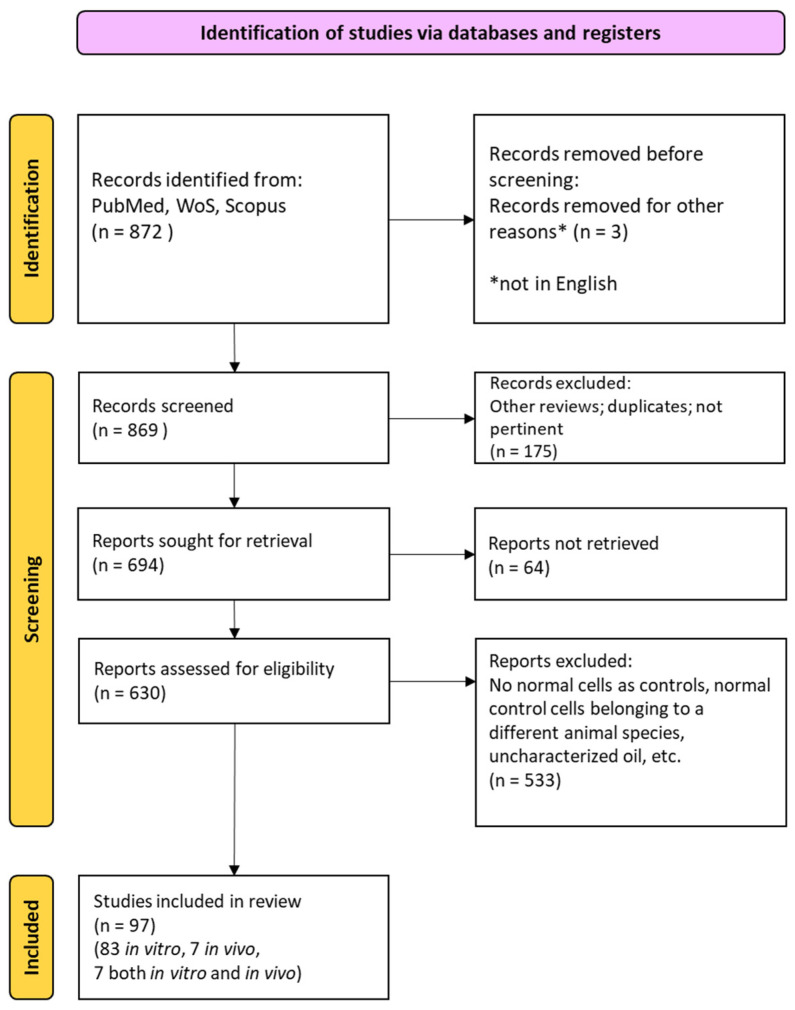
PRISMA flow diagram illustrating the study selection process.

The initial search yielded 872 studies, with 3 excluded due to non-English language. Screening of titles and abstracts from the remaining 869 studies led to the exclusion of 175 records, while 64 were unavailable, resulting in 694 full-text articles assessed for eligibility. Among these, 533 were excluded for the following reasons: use of uncharacterized oils, lack of non-tumor control cells, or controls derived from non-matching species. Ultimately, 97 studies (83 in vitro, 7 in vivo, and 7 using both approaches) were included in this systematic review. The whole selection process has been summarized in the following PRISMA flow diagram [20] (Figure 1).

### 3.1. Gastric Cancer

A total of 5 of the 97 selected studies investigated in vitro the anticancer activity of EOs from different plant species and individual EO constituents in gastric cancer models, using two human gastric cancer cell lines, HGC-27 and AGS. Overall, the tested EOs and single compounds exhibited selective cytotoxic or antiproliferative activity in gastric cancer cells through distinct mechanisms. Apoptosis was reported in 4 out of 5 studies, whereas cell-cycle arrest was observed in 2 studies and ROS-related mechanisms in 1 study.

The EO of *Micromeria congesta* showed strong cytotoxic activity in HGC-27 cells, inducing apoptosis via caspase activation and reducing the expression of matrix metalloproteinases MMP-2 and MMP-9 [21]. *Pistacia vera* (cv. Ohadi) hull EO inhibited the Wnt/β-catenin signaling pathway in AGS cells, while *Origanum onites* L. EO exerted selective cytotoxic effects associated with apoptosis, S and G2/M cell-cycle arrest, DNA double-strand breaks, and epigenetic modulation [22,23]. 

Among individual EO components, carvacrol and 3-carene showed selective antiproliferative activity in AGS cells. Carvacrol induced apoptosis through a pro-oxidant mechanism involving intracellular reactive oxygen species generation and glutathione depletion [24], whereas 3-carene triggered mitochondrial-driven apoptosis and G0/G1 cell-cycle arrest [25]. 

An overview of the in vitro studies discussed in this section is provided in Table 1.

No in vivo studies investigating EOs or their constituents in gastric cancer models were identified.

### 3.2. Skin Cancer

5 of the 97 selected studies investigated in vitro the anticancer activity of EOs in skin cancer models, including melanoma, squamous cell carcinoma, and basal cell carcinoma. These studies evaluated plant-derived EOs and EO formulations in different experimental systems, using established melanoma and squamous cell carcinoma cell lines as well as primary basal cell carcinoma cultures. ROS-associated mitochondrial dysfunction was reported in 2 out of 5 studies, whereas apoptosis and modulation of cancer-related signaling pathways were each observed in 1 study. In melanoma and squamous cell carcinoma models, *Syzygium aromaticum* EO exhibited selective cytotoxic activity in RPMI-7951 melanoma and A431 squamous cell carcinoma cells. These effects were associated with a marked increase in intracellular reactive oxygen species, mitochondrial dysfunction, and loss of membrane integrity, with eugenol identified as the major contributor to the observed bioactivity [26]. Similar mitochondria-driven mechanisms were reported for *Cedrus atlantica* and *Cymbopogon citratus* EOs using A375 melanoma cells, in which selective cytotoxicity was linked to caspase-3/7 activation, mitochondrial membrane depolarization, impairment of oxidative phosphorylation, and increased ROS production [27,28]. Additional evidence emerged from keratinocyte-derived models of skin carcinogenesis. In benign and malignant keratinocyte cell lines, *Lavandula vera* and *Salvia fruticosa* EOs showed selective, dose-dependent cytotoxic activity, preferentially targeting transformed cells [29]. Finally, studies performed on primary basal cell carcinoma cultures demonstrated that *Thymus serpyllum* and *Mentha × piperita* EOs exerted selective cytotoxic and antiproliferative effects, inhibiting clonogenicity, spheroid formation, and cell migration through modulation of Hedgehog and Notch signaling pathways [30].

An overview of the in vitro studies discussed in this section is provided in Table 2.

No in vivo studies investigating EOs or their constituents in skin cancer models were identified.

### 3.3. Breast Cancer

A total of 24 of the 97 selected studies investigated the in vitro anticancer activity of EOs, EO-based formulations, or their constituents in breast cancer models.

These studies mainly employed the estrogen receptor-positive MCF-7 cell line, the highly invasive triple-negative MDA-MB-231 model, the luminal T47D line, and the murine 4T1 breast cancer cells, allowing assessment of EO activity across different cellular subtypes with different levels of aggressiveness. Apoptosis was reported in 17 out of 24 studies, whereas cell-cycle arrest was observed in 11 studies and ROS-related mechanisms in 6 studies. Anti-migratory and anti-invasive effects were additionally reported in 5 studies. In MCF-7 cells, EOs from botanically diverse species—including *Tarchonanthus camphoratus*, *Meriandra dianthera*, and *Pallenis spinosa*—induced marked growth inhibition accompanied by G1/S or G0/G1 cell-cycle arrest and activation of apoptotic signaling, as demonstrated by Annexin V positivity, Bax upregulation, Bcl-2 downregulation, caspase activation, and PARP cleavage [31,32,33]. Comparable effects were also observed for EO nanoemulsions, such as those based on *Ferula assa-foetida* and *Zataria multiflora*, which in some cases showed enhanced cytotoxic potency or additional anti-angiogenic activity via VEGF-related signaling modulation [34,35].

Other key insights on the anticancer activity of EOs were provided by studies combining two-dimensional cultures with three-dimensional spheroid models. In this experimental context, the EOs of *Otanthus maritimus* and *Seseli tortuosum* emerged as the two most potent among six dune plant EOs initially screened and were further investigated for functional endpoints, demonstrating selective antiproliferative activity in both 2D and 3D systems, apoptosis induction (via PARP cleavage and sub-G1 accumulation), p21 upregulation, and suppression of the pro-survival AKT signaling [36].

In the MDA-MB-231 model, several EOs and EO-based formulations demonstrated selective cytotoxicity together with anti-migratory or anti-invasive effects. These outcomes were associated with the activation of the apoptotic cascade mediated by Bax/Bcl-2 modulation, caspase activation, mitochondrial dysfunction, and oxidative stress-related mechanisms, including ROS generation and activation of stress-response pathways such as Nrf2/HO-1 [37,38]. Nanoemulsions of *Heracleum persicum* and *Z. multiflora* EOs further highlighted the capacity of these formulations to enhance cytotoxicity and interfere with migration, cell-cycle progression, or DNA integrity in these highly invasive breast cancer cells [35,39].

Additional models supported the general relevance of these findings. In T47D cells, *Z. multiflora* EO nanoemulsion displayed exceptionally high potency, whereas Artemisia serotina EO exerted a predominantly cytostatic effect linked to G2/M arrest and redox imbalance [35,40]. In the murine 4T1 model, *Oliveria decumbens* EO induced robust apoptotic cell death characterized by ROS accumulation, mitochondrial membrane potential loss, caspase-3 activation, and extensive DNA fragmentation, confirming the activity of this EO also in highly aggressive breast cancer cells [41].

Finally, selected individual EO constituents—including carvacrol, thymol, carotol, δ-cadinene, and myristicin—resembled several cytotoxic and pro-apoptotic activities observed for the whole of the EOs, acting through ROS-associated mitochondrial dysfunction, caspase activation, cell-cycle arrest, and inhibition of migratory or invasive pathways [42,43,44,45].

An overview of the in vitro studies discussed in this section is provided in Table 3.

A total of 4 of the 97 selected studies investigated the in vivo anticancer efficacy of EOs or EO nanoformulations in breast cancer rodent models. These studies employed either chemically induced mammary carcinogenesis in rats or syngeneic 4T1 mammary carcinoma models in BALB/c mice, enabling the evaluation of EO activity in immunocompetent systems and under pathologically relevant conditions.

Across these models, in vivo administration of EOs or their nanoformulated derivatives consistently resulted in suppression of tumor growth, as evidenced by reductions in tumor volume, incidence, or weight. In chemically induced mammary cancer model, in mice, treatment with *Annona muricata* EO significantly reduced tumor burden, tumor multiplicity, and cumulative tumor volume, while increasing tumor latency and improving histopathological grade, effects associated with modulation of oxidative stress and angiogenic signaling, including decreased malondialdehyde and VEGF levels and increased glutathione content in cancer cells [55]. Similarly, administration of an incensole acetate nanoemulsion in a DMBA-induced breast cancer in rats resulted in reduced tumor volume and incidence, improved hematological parameters, enhanced antioxidant enzyme activity, and decreased synthesis of pro-inflammatory cytokines such as IL-1, IL-6, and TNF-α, together with increased apoptotic features in tumor tissue [51]. In syngeneic 4T1 breast cancer models, nanoencapsulated EOs consistently exerted dose-dependent antitumor effects. Nanoformulated cinnamon (*Cinnamomum cassia*) EO markedly inhibited tumor growth and tumor weight, reduced tumor cell proliferation as indicated by decreased Ki-67 expression, and increased apoptosis in tumor tissue, with nanoencapsulation clearly enhancing efficacy relative to the free EO [56]. Comparable findings were reported for nanoencapsulated frankincense (*Boswellia carterii*) EO, which significantly reduced tumor volume and induced extensive tumor necrosis while modulating cancer-related gene expression profiles, including downregulation of oncogenic drivers and upregulation of tumor suppressor genes, without inducing marked systemic toxicity [57].

Overall, although limited in number, the available in vivo studies indicate that EOs and their nanoformulated derivatives can suppress breast tumor growth through the combined modulation of tumor proliferation, apoptosis, oxidative stress, angiogenesis, and inflammatory environment, with nanoencapsulation consistently improving therapeutic outcomes.

An overview of the in vivo studies discussed in this section is provided in Table 4.

### 3.4. Lung Cancer

A total of 16 of the 97 selected studies investigated the in vitro anticancer activity of EOs, EO formulations, or individual EO constituents in lung cancer models. These studies employed a range of human lung cancer cell lines, including A549 and its subclone A-549-C5, NCI-H1299, NCI-H460 (including a drug-resistant variant), and H727 cells, allowing for the evaluation of EO activity across different cytological and molecular contexts. Apoptosis was reported in 11 out of 16 studies, whereas cell-cycle arrest and ROS-related mechanisms were each observed in 7 studies. Inhibition of migration and invasion was additionally reported in 3 studies.

In A549 cells, EOs and EO nanoemulsions from *Citrus limon*, *Croton tiglium*, *Oliveria decumbens*, and *Arachis* induced marked growth inhibition accompanied by apoptosis and cell-cycle arrest in lung cancer cells. These effects were associated with caspase-3 activation, mitochondrial dysfunction, redox imbalance, and modulation of apoptosis-related drivers, including modulation of the Bax/Bcl-2 ratio. Inhibition of migration and anti-angiogenic activity were also reported in specific experimental settings [44,58,59,60,61,62,63].

Individual EO constituents provided additional mechanistic insight. Geraniol exerted selective antiproliferative activity in A549 cells through inhibition of ornithine decarboxylase and hyaluronidase activities, accompanied by apoptosis induction, G2/M cell-cycle arrest, and disruption of tubulin polymerization [64]. Citronellol displayed antiproliferative effects in both A549 and NCI-H1299 cells by promoting necroptotic cell death mediated by TNF-α signaling, ROS accumulation, and RIP1/RIP3 pathway engagement, together with G1 phase cell-cycle arrest [65].

In NCI-H460 cells, mitochondrial metabolism and drug resistance emerged as relevant targets of EOs. Eugenol and *Syzygium aromaticum* EO formulations reduced cell viability in association with oxidative phosphorylation impairment, respiratory uncoupling, and oxidative stress [26]. Notably, nanoencapsulation strategies markedly enhanced cytotoxic efficacy. For example, 6,7-dehydroroyleanone, nanoencapsulated in hybrid nanoparticles, showed potent activity not only in parental NCI-H460 cells but also in the drug-resistant NCI-H460/R line, with cytotoxic effects independent of P-glycoprotein expression [66].

Finally, in H727 lung cancer cells, 3-carene demonstrated selective antiproliferative activity, inducing apoptosis associated with modulation of apoptosis-related markers and G0/G1 cell-cycle arrest [25].

An overview of the in vitro studies discussed in this section is provided in Table 5.

A total of 2 of the 97 selected studies investigated the in vivo anticancer efficacy of EO-based formulations or essential oils in lung cancer models. Both studies employed A549 human lung adenocarcinoma xenograft models in athymic nude mice, providing consistent evidence for the antitumor activity of EO compounds in vivo. Overall, the available evidence indicates significant tumor growth inhibition associated with apoptosis induction in tumor tissues. In a xenograft model, a carvacrol nanoemulsion administered orally at doses of 50 and 100 mg/kg induced a dose-dependent reduction in tumor weight (34.2% and 62.1%, respectively), accompanied by stabilization or recovery of body weight in treated animals. Mechanistic analyses supported apoptosis induction mediated by mitochondrial and oxidative stress-related pathways, consistent with in vitro findings [61]. Similarly, the essential oil of *Pittosporum glabratum*, administered intraperitoneally at 100 mg/kg, significantly reduced tumor volume and tumor weight in A549 xenograft models. Histological and TUNEL analyses confirmed increased apoptosis in tumor tissues, supporting a direct cytotoxic effect in vivo [67].

Collectively, these studies indicate that EO-based formulations and whole essential oils can exert antitumor effects in lung cancer models through mechanisms involving tumor growth inhibition and apoptosis induction in vivo.

An overview of the studies discussed in this section is provided in Table 6.

### 3.5. Colorectal Cancer (CRC)

A total of 17 of the 97 selected studies investigated the in vitro anticancer activity of EOs, EO-based formulations, or individual EO constituents in colorectal cancer models. These studies employed multiple human colorectal cancer cell lines, including HCT116 (p53 wild-type and p53-null variants), HCT-15, HT-29, RKO, Caco-2, SW-620, and LS-174-D3, enabling evaluation of EO activity across different genetic backgrounds and degrees of tumor aggressiveness.

Apoptosis was reported in 14 out of 17 studies, whereas cell-cycle arrest was observed in 5 studies and ROS-related mechanisms in 3 studies. In HCT116 cells, several EOs induced growth inhibition coupled to cell-cycle arrest and apoptosis, involving modulation of cell-cycle regulators, caspase activation, PARP cleavage, and suppression of pro-survival signaling pathways such as STAT3/JAK2, AKT, and ERK [44,59,71,72,73]. These multitarget effects were further highlighted by studies demonstrating redox-driven effects, including ROS-associated apoptosis and autophagy, SIRT1 inhibition, and p53-independent cell death in genetically distinct HCT116 models [74,75]. In addition, the EO of *Illicium verum* inhibited cell migration, invasion, and colony formation in HCT116 cells [76].

Additional colorectal cancer models supported the general relevance of these findings. In HT-29 and HCT-15 cells, EOs and EO nanoformulations induced selective cytotoxicity associated with mitochondrial dysfunction and caspase activation [27,48,77]. In RKO cells, essential oils from the sand-dune plants *Seseli tortuosum***,** *Otanthus maritimus***,** *Artemisia campestris*, and *Eryngium maritimum* displayed antiproliferative activity, with the most active extracts associated with p21 upregulation and apoptosis induction [36].

Individual EO constituents further refined these EO anticancer profiles. Eugenol and cinnamaldehyde exerted selective cytotoxic or antiproliferative activity in Caco-2 and SW-620 cells, inducing cell-cycle alterations and mixed apoptotic or necrotic cell death, with differential sensitivity between non-metastatic and metastatic models [78,79,80]. The compound 3-cyclohexene-1-methanol showed pronounced cytotoxicity in HT-29 and LS-174-D3 cells, associated with G2/M cell-cycle arrest, apoptosis, and inhibition of AKT and ERK1/2 signaling [81].

An overview of the in vitro studies discussed in this section is provided in Table 7.

A total of 3 of the 97 selected studies investigated the in vivo anticancer or chemopreventive activity of EOs or their constituents in colorectal cancer models, employing distinct experimental approaches that reflect different stages and drivers of tumor development.

One study addressed a therapeutic setting using a human colorectal cancer xenograft model. In ICR-SCID mice bearing HCT116 tumors, subcutaneous administration of terpinen-4-ol significantly inhibited tumor growth and was associated with increased apoptotic and oxidative stress markers within tumor tissue, without evidence of systemic toxicity, supporting a direct antitumor effect of this EO monoterpene [75].

In contrast, two studies focused on chemopreventive models of colorectal carcinogenesis. In genetically predisposed ApcMin/+ mice, oral administration of pogostone reduced intestinal polyp number and size, with ex vivo analyses of intestinal tissues indicating improved epithelial barrier integrity and modulation of immune responses and gut microbiota composition [83]. Similarly, in an inflammation-driven model of colon carcinogenesis, oral treatment with the EO of *Thymus hirtus* ssp. *algeriensis* inhibited tumor development, preserved colon architecture, and reduced histopathological damage, including tumor budding, in the absence of overt systemic toxicity, with mechanistic insights derived from ex vivo tissue analyses [72].

Collectively, the available in vivo evidence indicates that EOs and their bioactive constituents can exert both direct antitumor and chemopreventive effects in colorectal cancer animal models, with efficacy influenced by disease context and experimental design. Ex vivo molecular and histopathological analyses consistently support the involvement of apoptosis, inflammation control, and modulation of the intestinal microenvironment as key mechanisms of action.

An overview of the in vivo studies discussed in this section is provided in Table 8.

### 3.6. Hepatocellular Cancer

A total of 12 of the 97 selected studies investigated the in vitro anticancer activity of EOs, EO-derived formulations, or their constituents in hepatocellular carcinoma models. These studies were conducted predominantly in the HepG2 cell line, with additional evidence from Hep3B and Huh-7 models, and collectively indicate selective cytotoxic or antiproliferative effects of EO interventions in liver cancer cells. Apoptosis was reported in 5 out of 12 studies, whereas ROS-related mechanisms were observed in 1 study. Modulation of intracellular signaling pathways, including NF-κB and STAT3, was also reported across multiple studies.

In HepG2 cells, several whole EOs, including those from *Origanum vulgare*, *Croton matourensis*, *Anisosciadium lanatum*, *Chamaecyparis lawsoniana*, and *Rosmarinus officinalis*, exerted selective cytotoxic effects, often accompanied by apoptosis-related features such as phosphatidylserine externalization, DNA fragmentation, or modulation of apoptosis-associated markers including Bcl-2, NF-κB, caspase-3, and CYP-1A1 [48,84,85,86,87]. Individual EO constituents reproduced the key anticancer effects observed for their parent oils. Thymol and carvacrol reduced HepG2 cell viability in a concentration-dependent manner, while the sesquiterpene carotol displayed selective cytotoxic activity in the same model [44,84]. In several studies, in silico docking analyses were used in a complementary manner to suggest potential interactions between EO constituents and protein targets involved in cancer cell survival or apoptosis.

More advanced strategies highlighted the relevance of formulation and combination approaches. A nanoencapsulated formulation of *Origanum glandulosum* EO showed enhanced cytotoxic potency compared with the free EO [88], while a polyherbal combination of *Curcuma longa* and *Nigella sativa* EOs displayed cytotoxic activity comparable to 5-fluorouracil and greater efficacy than the individual oils or their major constituents [89].

Mechanistic depth was provided by one study in Hep3B cells, in which curcumol exerted selective antiproliferative effects through coordinated inhibition of STAT3 and HIF-1α signaling, involving suppression of p-STAT3 and reduced HIF-1α expression, and was associated with PD-L1 downregulation and reduced proliferative and angiogenic potential [90].

Collectively, the in vitro evidence indicates that EOs and their bioactive constituents can target hepatocellular carcinoma cells through multiple experimentally supported mechanisms, including direct cytotoxicity, apoptosis induction, modulation of survival and immune-related signaling pathways, and enhancement of efficacy through formulation or combination strategies. An overview of the studies discussed in this section is provided in Table 9.

A total of 2 of the 97 selected studies investigated the in vivo anticancer activity of EOs or their individual constituents in hepatocellular carcinoma models using human tumor xenografts in immunodeficient mice. Although limited in number, these studies provide complementary evidence for the antitumor potential of EO-based interventions in established hepatic tumors.

In an experimental setting, oral administration of curcumol significantly inhibited tumor growth in athymic nude mice bearing Hep3B tumors, without evidence of systemic toxicity. Tumor volume and weight reduction were accompanied by decreased expression of p-STAT3, HIF-1α, PD-L1, and VEGF in tumor tissues, as assessed ex vivo, supporting the involvement of immune- and angiogenesis-related pathways in the observed antitumor effects [90].

Consistent antitumor activity was also observed for the EO of *C. matourensis* in a HepG2 xenograft model established in C.B-17 SCID mice. Intraperitoneal treatment induced a dose-dependent reduction in tumor mass, reaching approximately 56%, without overt systemic toxicity, as indicated by unchanged body weight, organ weights, and analyzed hematological parameters [87].

Collectively, these in vivo studies indicate that EOs and their bioactive constituents can exert direct antitumor effects in hepatocellular carcinoma xenograft models, with tumor growth inhibition consistently supported by ex vivo molecular and histopathological analyses. An overview of the in vivo studies discussed in this section is provided in Table 10.

### 3.7. Oral and Hypopharyngeal Cancers

A total of 3 of the 97 selected studies investigated the in vitro anticancer activity of EOs or their constituents in oral and hypopharyngeal squamous cell carcinoma models. These studies employed the FaDu hypopharyngeal squamous cell carcinoma cell line and oral squamous carcinoma models, including KB and KON cell lines, providing initial evidence for the sensitivity of upper aerodigestive tract malignancies to EO-based interventions. Apoptosis was reported in 2 out of 3 studies, whereas cell-cycle arrest was observed in 1 study.

In hypopharyngeal squamous cell carcinoma cells, carvacrol exerted selective antiproliferative activity in FaDu cells, associated with cell-cycle arrest and apoptosis. These effects were linked to the inhibition of ornithine decarboxylase and hyaluronidase activities, enzymes involved in polyamine metabolism and extracellular matrix remodeling, highlighting metabolic and structural vulnerabilities in hypopharyngeal squamous cell carcinoma [93].

In oral squamous carcinoma cells, the EO of *Thymus caramanicus* demonstrated selective cytotoxic activity in KB cells, further supporting the susceptibility of oral epithelial malignancies to EO compounds [94]. Additional evidence from *Psidium guajava* leaf EO nanoemulsion demonstrated selective cytotoxic activity in KON oral cancer cells, associated with apoptosis induction and inhibition of colony formation and cell invasion [95].

Collectively, these in vitro studies indicate that EOs and their bioactive constituents can exert selective cytotoxic or antiproliferative effects in oral and hypopharyngeal squamous cell carcinoma models through mechanisms involving growth inhibition and apoptosis induction. An overview of the in vitro studies discussed in this section is provided in Table 11.

Only 1 of the 97 selected studies investigated the in vivo anticancer activity of an EO compound in oral squamous cell carcinoma. In this study, the monoterpenoid carvacrol, a major constituent of several EOs, was evaluated in a DMBA-induced oral squamous cell carcinoma model in rats, a well-established carcinogen-driven system that recapitulates key histopathological and molecular features of human oral cancer.

Topical administration of carvacrol, applied either concomitantly with DMBA exposure or following tumor induction, resulted in a marked suppression of oral carcinogenesis, as evidenced by reduced dysplastic and neoplastic lesions and improved histopathological architecture of the oral mucosa. These in vivo effects were supported by ex vivo immunohistochemical analyses, which revealed a significant reduction in the expression of PCNA, a marker of cell proliferation, and Bcl-2, an anti-apoptotic protein, indicating inhibition of tumor cell proliferation and activation of apoptotic pathways. Notably, carvacrol treatment was not associated with overt systemic toxicity, supporting its tolerability in this experimental setting.

Collectively, this in vivo evidence indicates that carvacrol can exert chemopreventive and therapeutic effects in oral squamous cell carcinoma, reinforcing the antiproliferative and pro-apoptotic activity observed in vitro and highlighting the potential relevance of EO compounds in oral cancer treatment [96].

### 3.8. Bladder Cancer

Only 1 of the 97 selected studies investigated the in vitro anticancer activity of EO compounds in bladder cancer models. This study evaluated the effects of the EO constituents carvacrol and thymol in human bladder carcinoma T24 cells, with the non-tumoral HEK-293 cell line used as a reference to assess selective cytotoxicity.

Both compounds exhibited selective, dose- and time-dependent antiproliferative activity in T24 cells. The observed growth inhibition was associated with mitochondrial dysfunction and activation of caspase-dependent apoptotic pathways, including PARP cleavage, together with cell-cycle arrest and reduced migratory capacity. These findings indicate that these EO monoterpenes can effectively interfere with key cellular processes involved in bladder cancer cell survival and progression [43].

No in vivo studies investigating EOs or their constituents in bladder cancer models were identified.

### 3.9. Cervical Cancer

A total of 6 of the 97 selected studies investigated the in vitro anticancer activity of EOs, EO-based formulations, or their individual constituents in cervical cancer models. These studies were conducted predominantly in the human cervical carcinoma HeLa cell line, with one study also employing the HeLa-R2 variant and the HEp-2 carcinoma model, as reported in the original study, providing a focused overview of EO activity across distinct mechanistic contexts in cervical cancer cells. Apoptosis was reported in 2 out of 6 studies, whereas cell-cycle arrest and ROS-related mechanisms were each observed in 1 study.

EOs from botanically diverse species, including *Erigeron canadensis*, *Orthosiphon schimperi*, *Myrtus communis*, and *Piper eriopodon*, induced marked reductions in HeLa cell viability, with apoptotic cell death supported by mitochondrial dysfunction, oxidative stress modulation, caspase activation, and PARP-1 cleavage in the studies where mechanistic endpoints were assessed [49,97,98,99].

Mechanistic complexity was further highlighted by studies investigating multi-component EO systems. The combined EOs of *Curcuma longa* and *Nigella sativa* exhibited enhanced cytotoxic potency compared with the individual oils and their major constituents, with complementary in silico analyses suggesting potential involvement of redox- and proliferation-related targets such as NOX2, NF-κB, and MDM2, consistent with a multitarget mode of action [89]. In parallel, *Thymus bovei* EO demonstrated selective cytotoxicity in the HeLa-R2 model, supporting the sensitivity of cervical cancer cells to EO compounds [59].

Collectively, these findings indicate that EOs and their bioactive constituents can exert direct cytotoxic effects in cervical cancer cell models through mechanisms involving mitochondrial dysfunction, oxidative stress, and caspase-dependent apoptosis, with multi-component formulations further enhancing anticancer efficacy.

An overview of the in vitro studies discussed in this section is provided in Table 12.

No in vivo studies investigating EOs or their constituents in cervical cancer models were identified.

### 3.10. Hematological Cancers

A total of 8 of the 97 selected studies investigated the in vitro anticancer activity of EOs or individual constituents in hematological cancer models. These studies were conducted in a range of human leukemia and lymphoma cell lines, including HL-60 and its drug-resistant variant HL-60R, Jurkat, Raji, K562, and MOLT-3, enabling assessment of EO activity across distinct hematological malignancies and resistance profiles.

Apoptosis was reported in 6 out of 8 studies, whereas cell-cycle arrest was observed in 3 studies and ROS-related mechanisms in 1 study. *Baccharis milleflora* EO exerted cytotoxic effects across multiple hematological cell lines, including HL-60, Jurkat, and Raji, characterized by induction of apoptotic and necrotic cell death and, in Raji cells, by G0/G1 cell-cycle arrest accompanied by a reduction in S and G2/M phases [100].

In acute myeloid leukemia models, *Glandora rosmarinifolia* EO showed selective cytotoxic activity in HL-60 cells and retained comparable efficacy in the drug-resistant HL-60R subline. In both models, apoptosis induction was associated with inhibition of topoisomerase II activity and G0/G1 cell-cycle arrest, supporting activity also in chemotherapy-resistant leukemia cells [101].

Similarly, the EO of *Ptychotis verticillata* exerted selective cytotoxic activity across multiple AML cell lines, including HL-60, HL-60R, KG-1, and K562, with cell death associated with oxidative stress and ROS-mediated apoptotic mechanisms. In the same study, the combination of carvacrol and thymol further enhanced cytotoxic effects and retained activity in drug-resistant cells [102].

In B-cell lymphoma (Raji cells), *Myrtus communis* EO displayed selective antiproliferative activity in Raji cells, indicating sensitivity of lymphoid malignancies to EO compounds [49].

In chronic myeloid leukemia K562 cells, EOs from *Alpinia galanga*, *Kelussia odoratissima* and *Varthemia iphionoides* demonstrated selective cytotoxic activity, with apoptosis induction reported for *Alpinia galanga* and *Varthemia iphionoides*, and dose-dependent loss of cell viability observed for *Kelussia odoratissima* [50,103,104]. Finally, the diterpenoid 6,7-dehydroroyleanone induced caspase-dependent intrinsic apoptosis in MOLT-3 acute lymphoblastic leukemia cells, characterized by marked sub-G1 accumulation and depletion of cells across all major cell-cycle phases [66].

An overview of the in vitro studies discussed in this section is provided in Table 13.

Only 1 of the 97 selected studies investigated the in vivo anticancer activity of individual EO constituents in hematological malignancies. In this study, a series of acyclic terpenoids commonly found in EOs, including citral, geraniol, nerol, and farnesol, were evaluated in a murine model of histiocytic lymphoma induced by U-937 cells.

All compounds were administered orally at a dose of 10 mg/kg, and antitumor efficacy was assessed in comparison with untreated controls and methotrexate as a reference chemotherapeutic agent. Treatment resulted in marked inhibition of lymph node growth, with citral showing the highest antitumor activity (≈88% inhibition), followed by geraniol (≈84%), nerol (≈75%), and farnesol (≈71%). Importantly, these antitumor effects were achieved without evidence of overt systemic toxicity, supporting the tolerability of EO-derived acyclic terpenoids in this experimental setting.

Collectively, this single in vivo study provides evidence that EO acyclic terpenoids can exert significant antitumor effects in hematological cancer models, complementing the cytotoxic and pro-apoptotic activity observed in vitro and supporting their further investigation as potential agents for hematological malignancies [106].

### 3.11. Prostate Cancer

3 of the 97 selected studies investigated the in vitro anticancer activity of EOs in prostate cancer models. These studies were conducted in androgen-independent human prostate carcinoma cell lines, namely DU145 and PC-3, providing initial evidence for the susceptibility of advanced prostate cancer cells to EO-based treatments.

Apoptosis and ROS-related mechanisms were each reported in 2 out of 3 studies. In DU145 cells, the EO of *Pinus mugo* significantly reduced cell viability and triggered apoptotic cell death, with mechanistic analyses indicating a central role for ROS-mediated oxidative stress and impairment of STAT3 signaling, a key pathway involved in prostate cancer cell survival [107].

Comparable cytotoxic effects were observed in PC-3 cells treated with the EO of *Hedychium spicatum*. In this model, EO exposure induced intrinsic mitochondrial apoptosis, characterized by ROS accumulation, loss of mitochondrial membrane potential, activation of caspase-3, -8, and -9, and modulation of Bcl-2 family proteins, leading to apoptotic cell death [108].

Collectively, these findings indicate that EOs can exert direct cytotoxic effects in prostate cancer cells through mechanisms involving oxidative stress, mitochondrial dysfunction, and activation of apoptotic pathways, supporting the relevance of EO compounds in prostate cancer research.

An overview of the in vitro studies discussed in this section is provided in Table 14.

No in vivo studies investigating EOs or their constituents in prostate cancer models were identified.

### 3.12. Ovarian Cancer

A total of 2 of the 97 selected studies investigated the in vitro anticancer activity of EO compounds in ovarian cancer models. These studies were conducted in human ovarian carcinoma cell lines, including PA-1, OAW 42, and SKOV3, providing evidence for the susceptibility of ovarian cancer cells to EO molecules and EO-based preparations.

In one study, the sesquiterpene β-caryophyllene, a common constituent of several EOs, exerted dose- and time-dependent antiproliferative activity in PA-1 and OAW 42 ovarian cancer cells. Growth inhibition was associated with apoptosis induction, mediated by caspase-3 activation and PARP cleavage, together with cell-cycle perturbation characterized by S-phase arrest, supporting a cytostatic and pro-apoptotic mode of action in ovarian cancer [109].

Complementary evidence was provided by a second study evaluating the EO of *Kelussia odoratissima* in the SKOV3 ovarian cancer cell line. In this model, EO exposure resulted in selective cytotoxic activity and a marked reduction in cell viability at higher concentrations, indicating sensitivity of epithelial ovarian cancer cells to EO complex mixtures [103].

Collectively, these in vitro studies indicate that EO constituents and EO-based preparations can exert direct cytotoxic or antiproliferative effects in ovarian cancer cell models through mechanisms involving apoptosis induction and cell-cycle disruption, warranting further investigation of EO-derived compounds in ovarian cancer research.

An overview of the in vitro studies discussed in this section is provided in Table 15.

No in vivo studies investigating EOs or their constituents in ovarian cancer models were identified.

### 3.13. Brain Cancer

A total of 3 of the 97 selected studies investigated the in vitro anticancer activity of EO compounds in brain cancer models, including human glioblastoma and neuroblastoma cell systems. These studies employed the T98, U251, and U87 glioblastoma models, together with the SH-SY5Y neuroblastoma cell line, providing insight into EO compound activity across distinct brain tumor contexts. Cell-cycle arrest and ROS-related mechanisms were each reported in 2 out of 3 studies, whereas apoptosis was observed in 1 study.

In one study, the sesquiterpene isofuranodiene, a constituent of *Smyrnium olusatrum* EO, exerted selective cytotoxic activity in T98, U251, and U87 glioblastoma cells. Growth inhibition was associated with G1-phase cell-cycle arrest and ROS-mediated necrotic cell death, indicating a prominent role of oxidative stress in the observed cytotoxic effects [110].

Complementary evidence was provided by a second study evaluating the combined activity of eugenol and β-caryophyllene in the U87 glioblastoma cell line. The combined treatment induced selective cytotoxic effects, characterized by reduced proliferation and migratory capacity, G0/G1 cell-cycle arrest, and transcriptional modulation of genes involved in cell-cycle regulation and invasion, supporting a synergistic, multitarget mode of action in glioblastoma cells [111].

In addition to glioblastoma models, a third study investigated the activity of the monoterpene myrcene in the SH-SY5Y neuroblastoma cell line. In this model, myrcene treatment resulted in selective cytotoxic activity associated with oxidative stress-related mitochondrial dysfunction and apoptosis induction, indicating that EO-derived monoterpenes can also target neuronal tumor cells beyond glioblastoma [112].

Collectively, these in vitro studies indicate that EO sesquiterpenes and monoterpenes can exert direct cytotoxic effects in brain tumor models through mechanisms involving oxidative stress, cell-cycle disruption, and cell-death pathways.

An overview of the in vitro studies discussed in this section is provided in Table 16.

No in vivo studies investigating EOs or their constituents in brain cancer models were identified.

### 3.14. Thyroid Cancer

Only 1 of the 97 selected studies investigated the in vitro anticancer activity of EO in a thyroid cancer model. This study evaluated the effects of the *Pistacia lentiscus* EO in the human follicular thyroid carcinoma FTC-133 cell line, using healthy fibroblasts as non-tumoral control cells to assess cancer selectivity.

Treatment with *P. lentiscus* EO resulted in a dose-dependent reduction in thyroid cancer cell viability while sparing normal fibroblasts, indicating selective cytotoxic activity. Growth inhibition was associated with a marked increase in intracellular reactive oxygen species (ROS) and activation of both intrinsic and extrinsic apoptotic pathways, as evidenced by caspase-8 and caspase-9 activation, mitochondrial membrane depolarization, and DNA fragmentation. Additional functional assays further demonstrated inhibition of clonogenic capacity, supporting a sustained antiproliferative effect in FTC-133 cells.

Collectively, this in vitro evidence indicates that *P. lentiscus* EO can exert ROS-dependent, apoptosis-mediated cytotoxic effects in follicular thyroid carcinoma cells, highlighting the sensitivity of this thyroid cancer subtype to EO-induced oxidative stress [113].

No in vivo studies investigating EOs or their constituents in thyroid cancer models were identified.

### 3.15. Sarcoma

Only 1 of the 97 selected studies investigated the in vitro anticancer activity of EO in a sarcoma model. This study evaluated the effects of the *Falcaria vulgaris* EO in the human liposarcoma SW-872 cell line, using normal human fibroblasts as non-tumoral controls to assess cancer selectivity.

Treatment with *F. vulgaris* EO resulted in a dose-dependent reduction in sarcoma cell viability, with increased sensitivity observed after longer exposure times. Flow-cytometric analyses demonstrated significant induction of cell death, involving both apoptotic and necrotic populations, while the underlying molecular mediators (e.g., caspase activation) were not fully characterized. Fractionation experiments further indicated that the most lipophilic fractions of the EO displayed the highest cytotoxic activity, suggesting a major contribution of highly hydrophobic EO constituents to the observed antitumor effects.

This in vitro study indicates that *F. vulgaris* EO can exert direct cytotoxic effects in liposarcoma cells through cell death-mediated mechanisms, highlighting the sensitivity of sarcoma cells to EO complex mixtures [114].

Only 1 of the 97 selected studies investigated the in vivo anticancer activity of EO in a sarcoma model. In this study, the EO of *Lippia microphylla*, characterized by high levels of thymol and carvacrol, was evaluated in a murine Sarcoma-180 transplantation model.

Intraperitoneal administration of *L. microphylla* EO resulted in significant inhibition of tumor growth in a dose-dependent manner compared with untreated controls. Tumor suppression was observed at both tested dose levels over a short treatment period, indicating measurable in vivo antitumor activity. Toxicological evaluation revealed moderate systemic toxicity, including transient alterations in hepatic and hematological parameters, which were reported as reversible and less severe than those induced by conventional chemotherapy in the same experimental setting.

Overall, this in vivo evidence indicates that *L. microphylla* EO can exert antitumor effects in an experimental sarcoma model, supporting the relevance of EO phenolic monoterpenes in sarcoma research [115].

## 4. Discussion

### 4.1. Cross-Cancer Comparative Analysis: Recurrent Patterns and Translational Gaps

When considered collectively, the tumor-specific evidence presented in this review reveals several recurrent biological and translational patterns that extend beyond individual cancer models.

Across nearly all tumor types examined, the most consistent mechanistic signature associated with EO exposure is the induction of oxidative stress-related mitochondrial dysfunction leading to regulated cell death. Increased ROS production, disruption of mitochondrial membrane potential, caspase activation, and modulation of Bcl-2 family proteins recur across gastric, breast, lung, colorectal, hepatocellular, brain, thyroid, hematological, and oral cancer models. Although the depth of mechanistic characterization varies between studies, this convergence suggests that redox imbalance represents a shared cellular vulnerability frequently exploited by EO constituents.

Cell-cycle perturbation constitutes a second recurrent feature. Arrest at G0/G1 or G2/M phases is reported across multiple tumor types and commonly accompanies apoptosis induction, indicating a coordinated growth-inhibitory response rather than isolated cytotoxicity. Subgroup analysis on more homogeneous datasets further supports this convergence. Phenolic monoterpenes such as carvacrol and thymol, tested across multiple cancer types, consistently induced apoptosis as the predominant biological response, frequently associated with oxidative stress, mitochondrial dysfunction, and caspase activation. Similarly, analyses within the same cellular models revealed coherent mechanistic patterns. In MCF-7 breast cancer cells, EOs from botanically distinct sources consistently triggered apoptosis, often accompanied by ROS generation, mitochondrial depolarization, and cell-cycle arrest. Comparable responses were observed in other widely used models, including A549 lung cancer and HCT116 colorectal cancer cells, supporting the presence of conserved EO-induced anticancer mechanisms across experimental contexts. In addition, nanoformulated EOs and EO-derived compounds generally exhibited enhanced cytotoxic effects compared to their non-formulated counterparts, likely reflecting improved cellular uptake and stability, and further reinforcing ROS-mediated mitochondrial dysfunction as a central mechanism.

From a translational perspective, important differences between tumor types emerge. Quantitatively, only 14 of the 97 included studies incorporated an in vivo component, and an even smaller fraction systematically evaluated therapeutic window or pharmacokinetic parameters. This numerical imbalance underscores the predominantly in vitro nature of current EO-based anticancer research and highlights a critical translational gap, as in vitro cytotoxicity alone is insufficient to predict therapeutic efficacy without pharmacokinetic, biodistribution, and safety validation. Breast and colorectal cancer models display the most consistent integration of in vitro and in vivo data, particularly in studies involving nanoformulated EO derivatives, where tumor growth inhibition is accompanied by molecular confirmation in tumor tissues. In contrast, for thyroid, bladder, ovarian, and prostate cancers, the available evidence remains largely restricted to in vitro systems, limiting the strength of translational inference.

Nanoformulation strategies recur across several solid tumor models and are frequently associated with enhanced cytotoxic potency and improved in vivo performance. However, heterogeneity in formulation design, dosing regimens, and pharmacokinetic assessment complicates direct comparison and prevents definitive conclusions regarding superiority over non-formulated compounds.

Certain tumor contexts highlight additional translational constraints. In brain tumor models, for example, cytotoxic activity is demonstrated in vitro without corresponding evidence of blood–brain barrier permeability. Similarly, across several hematological and solid tumor systems, demonstration of cytotoxic effects is not consistently accompanied by evaluation of therapeutic window or long-term toxicity.

Finally, specific EO constituents—including carvacrol, thymol, eugenol, and β-caryophyllene—recur across multiple tumor types and consistently display redox-associated cytotoxic and pro-apoptotic activity. This cross-tumor recurrence suggests that chemically defined EO constituents may offer greater translational consistency than chemically variable whole oils.

Overall, while EO research across cancer models shows notable mechanistic convergence, its translational maturity remains heterogeneous and strongly dependent on tumor type, formulation strategy, and experimental design. The studies included in this review do not always provide strong quantitative evidence of anticancer effects. However, the inclusion criteria were not intended to restrict the analysis to studies reporting standardized metrics, but rather to prioritize methodological rigor. In this context, studies lacking detailed potency measurements may still provide relevant biological insights when based on appropriate experimental design, whereas studies affected by methodological bias may generate misleading conclusions regardless of their apparent level of detail. This distinction is particularly relevant in EO research, where methodological variability represents a major source of inconsistency and limits the reliability of the evidence.

In fact, the high degree of methodological heterogeneity across studies substantially affects the possibility of drawing robust and generalizable conclusions, underscoring the need for standardized experimental approaches.

### 4.2. An Integrated View of the Antitumor Effect of EOs

EOs and their individual constituents exert antitumor activity through multiple, interconnected mechanisms, with selective cytotoxic and antiproliferative effects often linked to redox imbalance and cell death.

**Figure 2:**
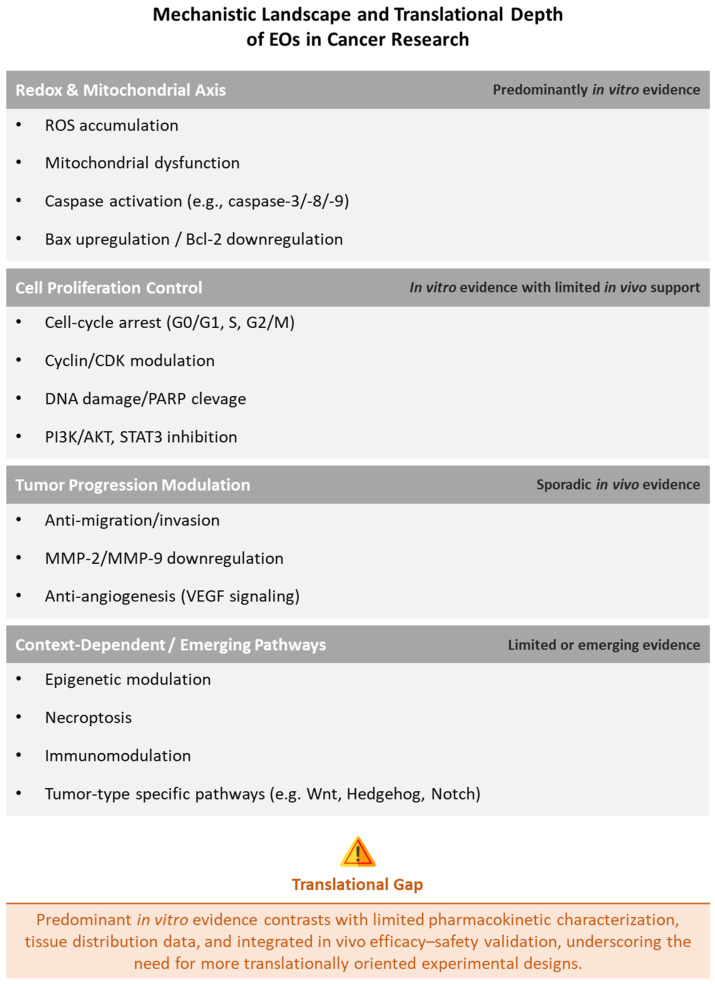
Mechanistic landscape and translational depth of essential oils (EOs) in cancer research.

The recurrent mechanistic convergence described above is schematically summarized in Figure 2.

The figure summarizes the recurrent biological mechanisms associated with EO exposure across tumor types, including redox and mitochondrial pathways, cell-cycle regulation, tumor progression modulation, and context-dependent mechanisms. It also highlights the prevailing translational gap between extensive in vitro mechanistic evidence and relatively limited pharmacokinetic characterization and integrated in vivo efficacy–safety validation.

Across different cancer types (e.g., gastric, breast, lung, hematological), many studies report that the central mechanism is an intracellular reactive oxygen species (ROS) accumulation, frequently associated with mitochondrial dysfunction, that involves membrane depolarization, cytochrome *c* release, and activation of the caspase cascade (e.g., caspase-3/-8/-9), together with a characteristic shift in Bcl-2 family signaling (Bax upregulation, Bcl-2 downregulation). In specific contexts, alternative death programs are also engaged; for example, in lung cancer models, citronellol has been associated with necroptosis via TNF-α/RIP1/RIP3 signaling.

At the same time, EOs and their molecules may inhibit proliferation by inducing cell-cycle arrest at G0/G1, S, or G2/M checkpoints through modulation of key regulators, including upregulation of inhibitors such as p21 and p53 and downregulation of cyclins (e.g., cyclin D1/cyclin B) and cyclin-dependent kinases (e.g., CDK1/CDK4), as documented in colorectal, breast, and cervical cancer models.

Beyond direct cytotoxicity, EOs modulate multiple oncogenic pathways. Suppression of pro-survival signaling (PI3K/AKT/mTOR and STAT3) is recurrent (e.g., breast, prostate, hepatocellular carcinoma), and inhibition of tumor-relevant pathways such as Wnt/β-catenin (gastric cancer) and Hedgehog/Notch (basal cell carcinoma) has also been reported. Modulation of pro-inflammatory mediators (e.g., IL-6, TNF-α) linked to tumor promotion is supported by in vivo studies. Anti-angiogenic effects have been evidenced by reduced VEGF signaling (in breast cancer in vivo), while anti-metastatic activity has been linked to decreased migration/invasion and downregulation of matrix metalloproteinase expression (e.g., MMP-2/MMP-9). In addition, several studies point to an induced metabolic vulnerability that involves the impairment of mitochondrial respiration/oxidative phosphorylation, the disruption of energy metabolism, and the increased oxidative stress responses.

At the molecular level, EO treatments have been associated with DNA damage (including double-strand breaks), inhibition of enzymes such as topoisomerase II and ornithine decarboxylase, and—based on in silico analyses—potential interactions with cancer-relevant receptors (e.g., ER-α, HER2). Epigenetic modulation (changes in promoter methylation and histone marks), that has been reported in specific models (e.g., gastric cancer), represents an additional EO target.

Overall, the antitumor activity of EOs reflects a complex network of complementary mechanisms converging on oxidative stress regulation, mitochondrial integrity, cell-cycle control, and oncogenic signaling, with context-dependent engagement of additional molecular targets depending on cancer types.

### 4.3. Nanoformulation Strategies to Enhance the Anticancer Potential of EOs

Based on the nanoformulation studies summarized in the Results tables, a recurrent limitation in the translational development of EOs and their constituents as anticancer agents is related to their unfavorable physicochemical and pharmacokinetic properties, including high volatility, low aqueous solubility, chemical instability, rapid systemic clearance, and limited bioavailability. To overcome these constraints and increase their therapeutic efficacy, a growing number of studies have explored nanoformulation and other delivery strategies aimed at improving EO stability, bioavailability and tumor targeting.

Across the included studies, the adoption of nanoemulsions, polymeric nanoparticles, lipid-based carriers, and hybrid nanosystems consistently demonstrated the ability to enhance the anticancer activity of EOs and their bioactive constituents compared with their free counterparts. Nanoformulation generally resulted in increased cellular uptake, improved dispersion in aqueous environments, and sustained release of lipophilic EO components, leading to lower effective concentrations and enhanced selectivity toward cancer cells, as consistently observed across multiple tumor models included in this review. In multiple in vitro models, nanoformulated EOs exhibited stronger antiproliferative and pro-apoptotic effects, often accompanied by augmented ROS accumulation, mitochondrial dysfunction, and caspase activation, while sparing non-tumoral cells.

Beyond efficacy enhancement, nanoformulations also addressed key issues related to tumor selectivity and safety. Several studies reported reduced off-target toxicity and improved tolerability profiles in vitro and also in vivo, with nanoformulated EOs frequently showing selective cytotoxicity toward cancer cells while sparing non-tumoral cells, supporting the notion that nanoencapsulation can widen the therapeutic window of EO-based interventions. This effect is particularly relevant for EO constituents characterized by narrow therapeutic margins when administered in free form, since nano-delivery systems can modulate biodistribution and mitigate peak-related toxicity.

Nanoformulation strategies also proved effective in overcoming pharmacological resistance mechanisms. The encapsulation of individual EO constituents into nanoparticle-based carriers enabled evasion of efflux transporter-mediated drug resistance, enhanced intracellular retention, and restored cytotoxic efficacy in multidrug-resistant cancer cell models, including hematological and solid tumors. In this context, nanocarriers function not only as passive delivery vehicles but also as active modulators of intracellular drug availability.

Collectively, these findings indicate that nanoformulation represents a critical enabling strategy for the advancement of EO-based anticancer approaches. By simultaneously improving stability, bioavailability, tumor targeting, and safety, nano-delivery systems help bridge the gap between promising in vitro activity and translational applicability, as partially supported by emerging in vivo evidence, particularly in studies involving nanoformulated EOs. Nevertheless, despite encouraging preclinical evidence, the heterogeneity of formulation designs, limited standardization, and scarcity of robust in vivo validation underscore the need for systematic optimization and comparative studies before nanoformulated EOs can be considered for clinical development.

### 4.4. Synergistic and Chemosensitizing Effects of EOs in Combination Therapies

A limited but mechanistically informative body of literature suggests that EOs and their individual constituents can exert synergistic or chemosensitizing effects when used in combination with other natural compounds or standard anticancer therapies. Although most of these studies were excluded from this review due to the absence of EO-alone arms, the non-standardized selectivity, or the exploratory experimental designs, the remaining ones provide relevant insight into combination-based strategies aimed at improving therapeutic efficacy and tolerability.

Clear examples of true molecular synergy have been reported for combinations of these constituents. The sesquiterpene β-caryophyllene and the phenylpropanoid eugenol, two major components of clove EO, displayed synergistic antitumor activity in U87 glioblastoma cells while sparing non-tumoral microglia (HMC3). Notably, each compound was largely ineffective when administered alone at the tested concentrations, whereas their combination induced apoptosis, G0/G1 cell-cycle arrest, and mitochondrial dysfunction, together with transcriptional downregulation of oncogenes (BCL2, MDM2, VEGFA), upregulation of tumor suppressors (TP53, PTEN), and reduced IL-4 secretion, indicating a combined cytotoxic and immunomodulatory effect [111]. Similarly, carvacrol and thymol, major components of oregano and thyme EO, exhibited strictly combination-dependent cytotoxicity in acute myeloid leukemia models (HL60, KG1, K562), including drug-resistant sublines, while sparing normal peripheral blood mononuclear cells. Mechanistically, the combined treatment triggered multi-pathway cell death involving apoptosis, oxidative stress, and endoplasmic reticulum stress; effects not observed with either compound alone thus supporting a real synergistic interaction [102].

In addition to EO–EO or compound–compound combinations, several studies explored chemosensitization of cancer cells to conventional anticancer drugs, particularly doxorubicin and 5-fluorouracil (5-FU). In hepatocellular carcinoma models, the treatment with *Silybum marianum* EO significantly enhanced the antitumor efficacy of 5-FU both in vitro and in vivo, resulting in reduced tumor growth, suppression of migration and invasion, modulation of Wnt/β-catenin and NF-κB signaling, inhibition of angiogenesis, activation of autophagy and apoptosis, and prolonged survival in an orthotopic mouse model [116]. Similar chemosensitizing effects have been reported for other EO constituents in combination with doxorubicin. For example, citral enhanced doxorubicin-induced cytotoxicity and apoptosis in human B-lymphoma cells while sparing normal peripheral blood mononuclear cells [117], whereas curcumol increased doxorubicin-mediated apoptosis and restored drug sensitivity in multidrug-resistant cancer cell lines [118]. Collectively, these findings suggest that EO molecules can re-sensitize resistant tumor cells by targeting stress-response, apoptotic, and survival pathways complementary to those engaged by standard chemotherapeutics.

A distinct subset of studies indicates that EOs may contribute to improving the therapeutic index of anticancer treatments, not by directly increasing tumor cytotoxicity, but by mitigating treatment-associated toxicity or enabling dose reduction in conventional drugs. Doxorubicin-induced cardiotoxicity—a major dose-limiting adverse effect linked to oxidative stress and mitochondrial damage—has been shown to be attenuated by specific EO constituents. Geraniol reduced doxorubicin-associated cardiotoxicity in experimental models, an effect attributed to its antioxidant and cytoprotective mechanisms [119]. In this context, *Thymus caramanicus* EO was shown to potentiate doxorubicin cytotoxicity at sub-effective drug concentrations in oral squamous carcinoma cells, supporting its role as an adjunctive agent capable of expanding the therapeutic window rather than acting as a primary anticancer treatment [94].

Finally, preliminary evidence suggests that EO constituents may also function as sensitizers in multimodal anticancer regimens. Myrcene, a monoterpenoid capable of interacting with DNA, enhanced radiation-induced damage in UV-irradiated cancer cells, indicating a potential radiosensitizing effect. However, such observations remain limited to in vitro models and require cautious interpretation and further validation [112].

Overall, the available evidence indicates that EOs and their constituents can participate in therapeutically relevant combination strategies through two complementary mechanisms: enhancement of antitumor efficacy via synergistic cytotoxicity or chemosensitization, and improvement of treatment tolerability through mitigation of dose-limiting toxicities. Nevertheless, the heterogeneity of experimental designs, frequent absence of formal synergy analyses, and limited in vivo validation underscore the need for rigorous preclinical frameworks before these approaches can be considered translationally relevant.

### 4.5. The Potential Role of EOs in Cancer Prevention and Chemoprotective Strategies

EOs and their constituents may exert biological activities relevant to cancer prevention, and their potential application in the healthcare sector has been mainly discussed in a preventive or supportive context. These compounds possess antioxidant, anti-inflammatory, antimutagenic, and antiproliferative properties, enabling them to modulate cancer-related pathways or to limit the cellular entry and bioactivation of mutagens [18].

EOs have been reported to modulate Phase I (e.g., CYP450) and Phase II (e.g., GST, NQO1) xenobiotic-metabolizing enzymes, thereby reducing the bioactivation of procarcinogens while enhancing their detoxification and elimination [120]. In addition to enzymatic modulation, EOs display pronounced antioxidant properties. Their ability to scavenge reactive oxygen species (ROS) plays a key role in limiting cellular oxidative damage, which represents a central mechanism in cancer prevention. This free-radical-scavenging activity contributes to the maintenance of cellular redox homeostasis and may reduce the risk of carcinogenesis [121,122].

Several EO constituents have been shown to influence cell fate decisions in premalignant or stressed cells by promoting apoptosis through caspase activation and by modulating the expression of regulatory proteins such as Bax and Bcl-2. Moreover, EOs can exert control over cell proliferation by inducing cell-cycle arrest at the G1/S and G2/M checkpoints, mediated by p21 and cyclin-dependent kinases (CDKs), and by inhibiting pro-survival signaling pathways, including mTOR/PKB, thereby potentially limiting neoplastic transformation [123].

Overall, this evidence derives predominantly from experimental and mechanistic studies and should be interpreted in the context of cancer prevention and supportive care rather than therapeutic intervention, reinforcing the distinction between preventive potential and the treatment-oriented strategies discussed in other sections of this review.

### 4.6. Bias and Limitations of Research Studies

A critical analysis of the available literature reveals recurrent methodological limitations that substantially undermine the reliability and translational relevance of many reported anticancer effects of EOs. Moreover, several studies draw strong conclusions based on limited mechanistic evidence or without adequate validation across independent models, increasing the risk of overstatement of therapeutic potential. This pattern is clearly reflected in the exclusion of 533 studies from the present review. Such exclusions were not arbitrary but instead arose from the systematic identification of three major and pervasive shortcomings: (i) the use of chemically uncharacterized EOs, (ii) the absence of appropriate normal cell controls to assess cancer selectivity, and (iii) the use of control cells derived from non-corresponding species or tissues. Together, these issues represent fundamental barriers to the development of evidence-based EO-dependent anticancer strategies.

Chemical characterization of EOs constitutes a non-negotiable prerequisite for meaningful biological interpretation. EOs are intrinsically complex and volatile mixtures, often containing up to 50 bioactive constituents whose relative abundance is strongly influenced by geographic origin, soil composition, climate, harvest time, plant part, extraction method, and storage conditions [8,17,124]. Consequently, two oils marketed under the same botanical name may display profoundly different chemical profiles and biological activities. Importantly, additional sources of variability are introduced by processing-related factors, including extraction methods, distillation parameters, storage conditions, as well as botanical aspects such as the specific plant part used. These factors further shape the compositional profile of EO mixtures and critically influence their bioactivity, and the lack of chemical standardization represents a major barrier to cross-study comparability and prevents the establishment of reliable structure–activity relationships, making experimental outcomes difficult to predict and reproduce when EO composition is not fully controlled and reported [125]. In the absence of detailed compositional analysis, biological findings cannot be reliably compared across studies, nor can structure–activity relationships be established. This limitation does not represent a minor reporting issue, but a fundamental flaw that prevents reproducibility and severely compromises the biological interpretation of EO-related effects.

In response to this intrinsic variability, a growing trend in the healthcare and translational research sectors has been to focus on the investigation of individual EO constituents rather than whole oils. This reductionist approach facilitates standardization (pharmacological grade molecules), quality control, and pharmacokinetic analyses, while enabling precise identification of molecular targets. Importantly, the use of single, well-defined molecules enhances the reliability and interpretability of both experimental findings and downstream clinical investigations [126]. While this strategy does not cancel the relevance of whole EOs experiments, it highlights the necessity of clearly defining the chemical entity under investigation when mechanistic or therapeutic claims are made.

An equally critical and frequently neglected limitation concerns the lack of appropriate normal cell controls. Despite widespread reports of EO-induced cytotoxicity in cancer cell lines, many studies fail to assess toxicity in non-malignant cells under comparable experimental conditions. Without such controls, claims of cancer selectivity remain fundamentally unsupported. Importantly, although EOs are widely used in traditional medicine, they are not inherently innocuous; several constituents can induce irritation, cytotoxicity, or tissue damage in a dose- and exposure-dependent manner [17,127].

This methodological gap has direct and clinically relevant implications: without comparative toxicity evaluation in appropriate normal cell models, it is impossible to distinguish genuine, tumor-selective cytotoxicity from non-specific cellular damage that would likely manifest as unacceptable side effects in vivo [128,129,130]. Therefore, the inclusion of suitable normal cell controls is not merely an optional validation step, but a fundamental requirement for establishing both the efficacy and tumor specificity of EO activities [130]. In this context, the absence of appropriate non-tumoral controls may lead to a systematic overestimation of anticancer selectivity, as general cytotoxic effects can be misinterpreted as tumor-specific activity.

Furthermore, when such controls are employed, their appropriate selection represents an equally critical yet frequently overlooked factor. To generate biologically meaningful and predictive data, control cells should derive not only from the same species (e.g., human) [131,132,133] but, wherever possible, from the same tissue of origin as the tumor model under investigation. This requirement for tissue compatibility is supported by extensive evidence demonstrating that anticancer agents, including conventional chemotherapeutics, often display highly tissue-specific toxicity profiles in normal cells. Neglecting this principle by relying on mismatched controls (e.g., dermal fibroblasts to assess agents targeting hepatic carcinoma) increases the risk of failing to detect clinically relevant off-target toxicity, thereby undermining claims of selective anticancer activity [134].

Failure to account for this dimension introduces substantial bias and limits the predictive value of in vitro findings. A paradigmatic illustration of these translational challenges is provided by the study [24]. In this work, carvacrol exhibited clear dose-dependent cytotoxic and pro-apoptotic effects in vitro, with greater sensitivity of human gastric adenocarcinoma cells compared to normal fibroblasts. However, prolonged oral administration of carvacrol in vivo failed to inhibit tumor growth and was instead associated with marked gastrointestinal toxicity and systemic oxidative stress, including glutathione depletion in healthy tissues. Importantly, the authors did not question the anticancer mechanism of carvacrol itself but highlighted its concentration-dependent pro-oxidant nature and the resulting narrow therapeutic window. This study exemplifies how well-designed in vitro investigations, when not supported by adequate pharmacokinetic and dose-optimization strategies, may fail to translate into safe and effective in vivo outcomes [24].

Translational limitations further arise from the frequent assumption that robust in vitro cytotoxicity will directly predict in vivo efficacy. In reality, systemic exposure, biodistribution, metabolism, and clearance critically determine whether EO constituents can reach the tumor at biologically relevant concentrations without inducing unacceptable body toxicity. These challenges are particularly evident in tumors protected by physiological barriers, such as central nervous system malignancies, where the blood–brain barrier severely restricts compound penetration [111]. In such contexts, basic pharmacokinetic and biodistribution data become indispensable, and their absence markedly reduces the biological relevance of in vitro observations [135].

For these reasons, the present review was intentionally designed not as an exhaustive catalog of EO-related anticancer studies, but as a critical appraisal aimed at identifying minimum methodological requirements for reliable, reproducible, and translationally meaningful research on EOs and their constituents. Without rigorous chemical characterization, appropriate normal and tissue-matched controls, and explicit consideration of pharmacokinetic constraints, even compelling anticancer activity observed in vitro risks remaining biologically ambiguous and clinically irrelevant. Establishing these minimum standards is therefore essential for EOs and their molecules to progress from experimental observations toward credible anticancer applications. An additional aspect that warrants consideration is the potential impact of publication bias. Studies reporting positive anticancer effects are more likely to be published, whereas negative or inconclusive findings are often underrepresented. This imbalance may contribute to an overestimation of the consistency and efficacy of EO-related anticancer activity across experimental models.

### 4.7. Defining Minimum Methodological Standards for EO-Based Anticancer Research

**Figure 3:**
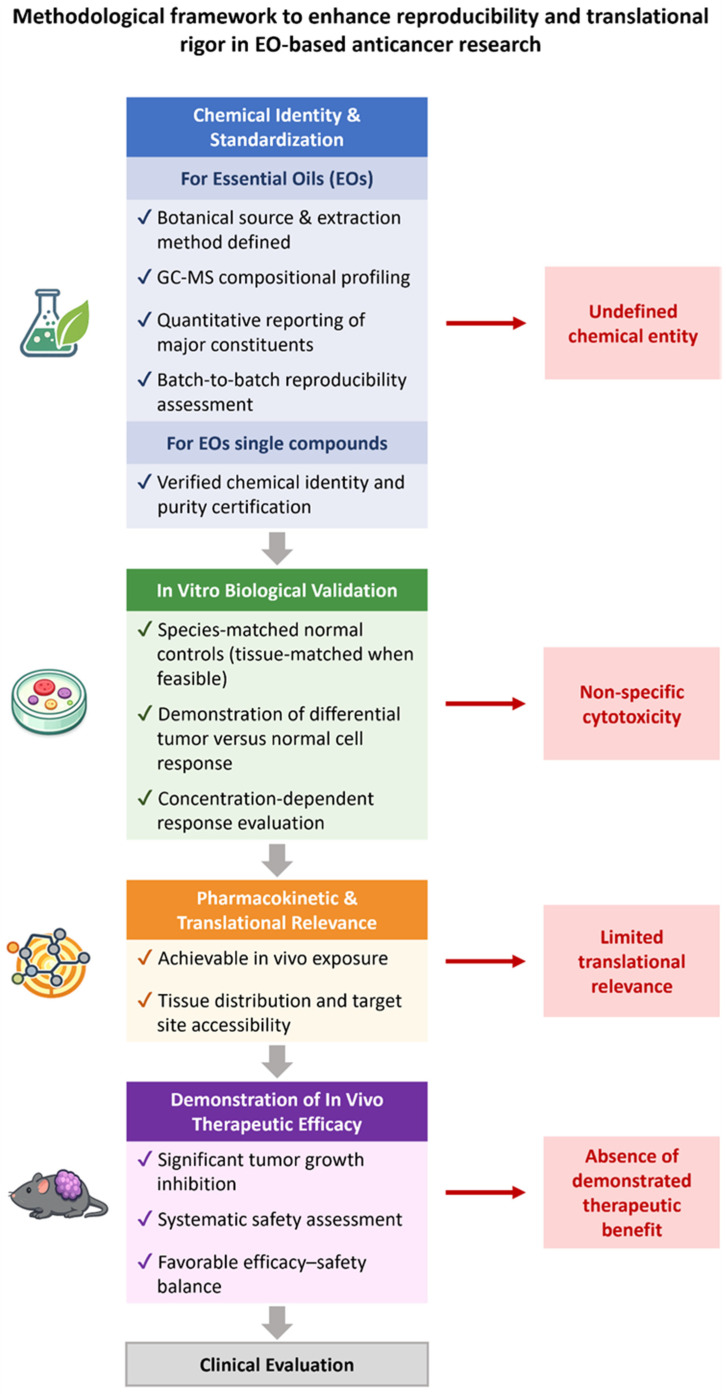
Methodological framework to enhance reproducibility and translational rigor in EO-based anticancer research.

In light of the methodological limitations outlined above, the advancement of EO-based anticancer research requires clearer and more consistent experimental standards. Greater methodological rigor is essential to improve reproducibility, cross-study comparability, and translational relevance. The proposed framework is illustrated in Figure 3.

The figure outlines the minimum experimental standards proposed to improve the design, reproducibility, and reporting of preclinical EO-based anticancer studies. Four interconnected domains are highlighted: (i) chemical identity and standardization, (ii) in vitro biological validation with appropriate controls, (iii) pharmacokinetic and translational relevance, and (iv) in vivo therapeutic efficacy and safety evaluation. The lower panel highlights recurrent methodological pitfalls—including undefined chemical entities, non-specific cytotoxicity, and limited translational relevance—that the proposed framework aims to address.

## 5. Conclusions and Future Perspectives

Many studies have been published regarding the antitumor properties of EOs. Most of these, however, have overlooked critical variables and potential biases in their experimental design, weakening their conclusions. This review critically evaluates the anticancer effects of EOs and their bioactive constituents by focusing exclusively on studies that rigorously address these limitations through meticulous experimental design. In addition to synthesizing these robust findings, we provide concrete recommendations to enhance methodological rigor in future research on EOs. Given the rapidly expanding yet heterogeneous body of literature on these natural compounds, the adoption of such stringent quality criteria is essential to establish reliable structure–activity relationships and to strengthen the translational potential of EO-derived anticancer agents.

## Figures and Tables

**Table 1:** In vitro studies with EOs and single compounds on gastric cancer cell lines.

Cell Line	EO/Single Compound	IC_50_	Effects	Reference
HGC-27	EO of *Micromeria congesta*	15.84 µg/mL (MTT, 24 h)	Selective cytotoxic activity. Induction of apoptosis (caspase-3 and caspase-8 upregulation) and downregulation of MMP-2 and MMP-9 expression.	[21]
AGS	Carvacrol	82.57 µM (CellTiter-Glo, 24 h)	Selective cytotoxic activity. Induction of apoptosis associated with increased intracellular ROS generation, glutathione depletion, and DNA damage.	[24]
AGS	EO of *Pistacia vera* (cv. Ohadi) hull	195 µg/mL (MTT, 24 h)	Inhibition of the Wnt/β-catenin signaling pathway and downregulation of Wnt-related proliferative genes.	[22]
AGS	EO of *Origanum onites* L.	28.23 µg/mL (WST-8, 24 h)	Selective cytotoxic activity. Induction of apoptosis, S and G2/M cell-cycle arrest, and DNA double-strand breaks, associated with epigenetic remodeling, including reduced promoter methylation of tumor suppressor genes and alterations in global and gene-specific histone modifications.	[23]
AGS	3-Carene	12.30 µg/mL (MTT, 48 h)	Selective, dose-dependent antiproliferative activity. Induction of apoptosis and G0/G1 cell-cycle arrest associated with mitochondrial apoptotic signaling.	[25]

**Table 2:** In vitro studies with EOs and single compounds on skin cancer cell lines.

Cell Line	EO/Single Compound	IC_50_	Effects	Reference
RPMI-7951 (melanoma)	*Syzygium aromaticum* EO (formulated with Tween-20/PEG-400)	N/A	Selective cytotoxic activity. Increased intracellular ROS production and mitochondrial dysfunction associated with loss of membrane integrity and increased LDH release.	[26]
A431 (squamous cell carcinoma)	*Syzygium aromaticum* EO (formulated with Tween-20/PEG-400)	N/A	Selective cytotoxic activity. Increased ROS production and mitochondrial dysfunction, with a predominant contribution of eugenol.	[26]
A375 (melanoma)	EO of *Cedrus atlantica*	N/A	Selective cytotoxic activity. Induction of mitochondria-mediated apoptosis associated with caspase-3/7 activation, loss of mitochondrial membrane potential, and inhibition of oxidative phosphorylation.	[27]
A375 (melanoma)	EO of *Cymbopogon citratus*	N/A	Selective cytotoxic activity. Increased intracellular ROS production, mitochondrial membrane depolarization, and impaired mitochondrial respiratory function, indicating mitochondrial dysfunction; supported by in silico analyses.	[28]
A5 (benign keratinocytes)	EO of *Lavandula vera*	N/A	Selective cytotoxic activity	[29]
A5 (benign keratinocytes)	EO of *Salvia fruticosa*	N/A	Selective cytotoxic activity	[29]
II4 (malignant keratinocytes)	EO of *Lavandula vera*	N/A	Selective cytotoxic activity	[29]
II4 (malignant keratinocytes)	EO of *Salvia fruticosa*	N/A	Selective cytotoxic activity	[29]
Primary basal cell carcinoma (BCC) cells	EO of *Thymus serpyllum* L.	262 µg/mL (MTT, 72 h)	Selective cytotoxic activity. Inhibition of clonogenicity, spheroid formation, and migration, associated with downregulation of Hedgehog pathway components (SMO, GLI1).	[30]
Primary basal cell carcinoma (BCC) cells	EO of *Mentha × piperita* L.	556 µg/mL (MTT, 72 h)	Selective cytotoxic activity accompanied by antiproliferative effects. Reduced clonogenic and spheroid-forming capacity and migration, associated with modulation of Hedgehog (PTCH1) and Notch (Notch1, JAG1) signaling pathways.	[30]

**Table 3:** In vitro studies with EOs and single compounds on breast cancer cell lines.

Cell Line	EO/Single Compound	IC_50_	Effects	Reference
MCF-7	EO of *Tarchonanthus camphoratus*	12.5 µg/mL (MTT, 24 h)	Selective cytotoxic activity. Cell-cycle arrest at G1/S phase. Induction of early and late apoptosis (Annexin V/PI). Upregulation of p53, Bax, and caspases-9, -8, -3; downregulation of Bcl-2.	[32]
MCF-7	EO of *Psidium guajava*	N/A	Selective cytotoxic activity. In silico docking suggests interaction of major sesquiterpenes (caryophyllene, caryophyllene oxide) with ER-α ligand-binding domain, supporting a potential SERM-like mechanism.	[46]
MCF-7	*Ferula assa-foetida* EO Nanoemulsion	64.42 µg/mL (MTT, 48 h)	Selective cytotoxic activity. Induction of apoptosis confirmed by AO/PI staining. Upregulation of Bax and downregulation of Bcl-2. Anti-angiogenic activity via downregulation of VEGF and VEGFR.	[34]
MCF-7	Myristicin	N/A	Selective cytotoxic activity. G1/S cell-cycle arrest, associated with Cdk1 downregulation. Induction of apoptosis associated with increased ROS levels, activation of caspase-8 and caspase-9, upregulation of Bax and p53, downregulation of Bcl-2, PARP cleavage, and inhibition of cell migration and glucose uptake.	[45]
MCF-7	EO of *Meriandra dianthera*	83.6 µg/mL (MTT, 48 h)	Selective cytotoxic activity. Induction of apoptosis confirmed by Annexin V/PI flow cytometry and morphological alterations.	[31]
MCF-7	EO of *Pallenis spinosa* (flower)	0.25 µg/mL (flow cytometry-based viability assay, 48 h)	Selective cytotoxic activity. Induction of caspase-dependent and caspase-independent apoptosis, associated with Bax upregulation and Bcl-2 downregulation. G0/G1 cell-cycle arrest mediated by modulation of cyclin D1, CDK4, and p21 expression. Leaf EO showed the same mechanisms but lower cytotoxic potency (IC_50_ = 2.4 µg/mL).	[33]
MCF-7	EO of *Otanthus maritimus*	0.21 µL/mL (MTS, 72 h)	Selective antiproliferative activity in 2D and 3D breast cancer models. Induction of apoptosis, evidenced by increased PARP cleavage and sub-G1 population, accompanied by p21 upregulation and downregulation of pro-survival AKT signaling. Among six dune plant EOs tested, this EO was one of the two most potent and was further investigated for functional endpoints.	[36]
MCF-7	EO of *Seseli tortuosum*	0.0086 µL/mL (MTS, 72 h)	Selective antiproliferative activity in 2D and 3D breast cancer models. Induction of apoptosis, evidenced by increased PARP cleavage and sub-G1 population, accompanied by p21 upregulation and downregulation of pro-survival AKT signaling. Among six dune plant EOs tested, this EO was one of the two most potent and was further investigated for functional endpoints.	[36]
MCF-7	*Zataria multiflora* EO nanoemulsion	5.38 µg/mL (MTT, 72 h)	Selective cytotoxic activity. Morphological changes and AO/EB staining indicative of apoptosis.	[35]
MCF-7	Carvacrol nanoencapsulated	7.0 µg/mL (MTT, 48 h)	Marked enhancement of selective cytotoxic activity compared to free carvacrol (~12-fold IC_50_ reduction).	[47]
MCF-7	Carvacrol	26.76 µg/mL (MTT, 72 h)	Selective antiproliferative activity. ROS-mediated mitochondrial apoptosis, caspase activation, PARP cleavage, cell-cycle arrest, and inhibition of migration.	[43]
MCF-7	Thymol	27.11 µg/mL (MTT, 24 h)	Selective antiproliferative activity. Induction of apoptosis associated with increased ROS levels, mitochondrial dysfunction, caspase activation, PARP cleavage, cell-cycle arrest, and reduced migratory capacity.	[43]
MCF-7	Carotol	41.73 µM (MTT, 24 h)	Selective cytotoxic activity.	[44]
MCF-7	EO of *Rosmarinus officinalis*	1.72 µg/mL (MTT, 72 h)	Selective cytotoxic activity.	[48]
MCF-7	EO of *Myrtus communis*	19.59 µg/mL (MTT, 72 h)	Selective antiproliferative activity.	[49]
MCF-7	EO of *Varthemia iphionoides*	188.8 µg/mL (MTT, 24 h)	Selective cytotoxic activity.	[50]
MDA-MB-231	*Heracleum persicum* EO nanoemulsion (anethole-rich)	2.32 µg/mL (MTT, 48 h)	Selective cytotoxic activity. Inhibition of cell migration. Induction of apoptosis, evidenced by caspase-3 upregulation and an increased sub-G1 cell cycle population.	[39]
MDA-MB-231	EO of *Pallenis spinosa flower*	0.21 µg/mL (flow cytometry-based viability assay, 48 h)	Selective cytotoxic activity. Induction of caspase-dependent and caspase-independent apoptosis, associated with Bax upregulation and Bcl-2 downregulation. G0/G1 cell-cycle arrest mediated by modulation of cyclin D1, CDK4, and p21 expression. Leaf EO displayed similar mechanisms with lower cytotoxic potency (IC_50_ = 1.5 µg/mL).	[33]
MDA-MB-231	EO of *Decatropis bicolor*	53.81 µg/mL (MTT, 24 h)	Selective cytotoxic activity. Induction of apoptosis associated with Bax upregulation, Bcl-2 downregulation, activation of caspase-9 and caspase-3, DNA fragmentation, and apoptotic morphological changes.	[38]
MDA-MB-231	EO of *Thymus vulgaris*	75.1 µg/mL (SRB, 24 h)	Selective cytotoxic activity. Inhibition of cell migration and colony formation. Induction of apoptosis associated with increased Bax/Bcl-2 ratio, mitochondrial membrane potential loss, and caspase-3 cleavage. Modulation of apoptosis-related proteins (IAPs) and oxidative stress response, including early ROS generation and activation of the Nrf2/HO-1 pathway.	[37]
MDA-MB-231	Incensole acetate nanoemulsion	N/A	Selective cytotoxicity. In silico docking predicts binding to key breast cancer receptors: Estrogen Receptor (ER), Progesterone Receptor (PR), and HER2, with the highest affinity for ER.	[51]
MDA-MB-231	*Zataria multiflora* EO nanoemulsion	20.4 µg/mL (MTT, 72 h)	Selective cytotoxic activity. ROS-mediated mitochondrial apoptosis (ΔΨm loss, Annexin V+, DNA fragmentation); G2/M cell cycle arrest (2D); DNA damage and direct DNA interaction.	[35]
MDA-MB-231	δ-Cadinene	1.7 µM (MTT, 24 h)	Selective cytotoxic activity. Inhibition of cell invasion associated with reduced MMP-2 activity and modulation of adhesion- and invasion-related pathways.	[42]
MDA-MB-231	EO of *Juniperus chinensis*	38 µg/mL (XTT, 72 h)	Selective cytotoxic activity.	[52]
MDA-MB-231	2-carene nanoemulsion	N/A	Selective cytotoxic activity. Inhibition of cancer cell proliferation. Induction of apoptosis.	[53]
T47D	*Zataria multiflora* EO nanoemulsion	0.0016 µg/mL (MTT, 72 h)	Strong selective cytotoxicity; highest sensitivity among tested breast cancer cell lines; marked enhancement compared to free EO.	[35]
T47D	EO of *Artemisia serotina* Bunge	40.81 µg/mL (MTT, 24 h)	Selective cytotoxic activity. Predominantly cytostatic effect associated with G2/M cell-cycle arrest and disturbance of intracellular redox balance, with moderate induction of apoptosis.	[40]
T47D	EO of *Chiliadenus iphionoides*	0.08 mg/mL (MTT, 48 h)	Selective cytotoxic activity.	[54]
4T1	EO of *Oliveria decumbens*	47.3 μg/mL (MTT, 24 h)	Selective cytotoxic activity. Induction of apoptosis evidenced by morphological changes, AO/EB staining, Annexin V positivity, and caspase-3 activation. Increased ROS levels, loss of mitochondrial membrane potential (ΔΨm), and DNA fragmentation and damage (laddering and comet assay).	[41]

**Table 4:** In vivo studies with EOs and single compound formulations in breast cancer models.

Model	EO/Single Compound	Dosage	Effects	Reference
Female Sprague-Dawley rats (DMBA-induced mammary carcinoma)	Incensole acetate nanoemulsion	50 mg/kg, intragastric (every other day for 4 weeks)	Reduced tumor volume and tumor incidence. Improvement of hematological parameters (WBCs, platelets). Modulation of oxidative stress markers, including increased catalase and glutathione peroxidase activity. Reported attenuation of pro-inflammatory cytokines (IL-1, IL-6, TNF-α). Increased apoptotic features in tumor tissue	[51]
4T1 murine mammary carcinoma (BALB/c mice)	*Cinnamon cassia* EO nanoencapsulated	6.25, 12.5, 25 mg/kg (oral, 14 days)	Dose-dependent inhibition of tumor growth and tumor weight. Increased tumor cell apoptosis (TUNEL assay). Reduced tumor cell proliferation, evidenced by decreased Ki-67 expression. Nanoencapsulated EO markedly more effective than free EO (tumor inhibition ≈ 44.6% at 25 mg/kg).	[56]
4T1 murine mammary carcinoma (BALB/c mice)	*Boswellia carterii* EO nanoencapsulated	12 µL/g, oral administration (21 days)	Significant reduction in tumor volume and extensive tumor necrosis. Enhanced antitumor efficacy of the nanoformulated EO compared with the free EO, without marked systemic toxicity. In vivo modulation of cancer-related gene expression, including downregulation of oncogenes (PIK3CA, CCND1, HER2, STAT3, KRAS) and upregulation of tumor suppressor genes (TP53, CDH1, PTEN).	[57]
Female rats (DMBA-induced mammary carcinoma)	EO of *Annona muricata*	50, 100, 200 mg/kg/day, oral administration (13 weeks)	Dose-dependent reduction in tumor incidence, tumor multiplicity, and cumulative tumor volume (≈50–71%). Increased tumor latency and improvement of histopathological grade (III to I). Modulation of oxidative stress and angiogenic signaling, including decreased malondialdehyde and VEGF levels and increased reduced glutathione content.	[55]

**Table 5:** In vitro studies with EOs and single compounds on lung cancer cell lines.

Cell Line	EO/Single Compound	IC_50_	Effects	Reference
A549	*Citrus lemon* EO Nanoemulsion	31.65 µg/mL (MTT, 24 h)	Selective cytotoxic activity. Induction of apoptosis via caspase-3 upregulation. Anti-angiogenic activity in CAM assay.	[63]
A549	EO of *Croton tiglium*	48.38 µg/mL (CCK-8, 48 h)	Selective antiproliferative activity. Cell-cycle disruption via downregulation of cyclin A, cyclin B, and CDK1. Induction of mitochondrial apoptosis with loss of MMP and caspase activation. Inhibition of migration.	[62]
A549	EO of *Oliveria decumbens*	22.14 µg/mL (MTT, 24 h)	Selective cytotoxic activity (vs. L929). ROS-mediated apoptosis with increased caspase-3 activity and modulation of Bax/Bcl-2 ratio.	[60]
A549	*Arachis hypogaea* EO Nanoemulsion	7.21 µg/mL (MTT, 24 h)	Selective cytotoxic activity (vs. HFF). Caspase-3-mediated apoptosis associated with Sub-G1 cell-cycle arrest.	[58]
A549	Carvacrol Nanoemulsion	N/A	Selective cytotoxic activity. Mitochondrial apoptosis associated with increased ROS production and modulation of apoptosis-related markers, including JNK phosphorylation, Bax upregulation, Bcl-2 downregulation, cytochrome-c release, and caspase activation.	[61]
A549	Geraniol	91.44 µM (SRB, 72 h)	Selective antiproliferative activity. Inhibition of ornithine decarboxylase (ODC; IC_50_ = 15.42 µM) and hyaluronidase (IC_50_ = 57.61 µM) activities. Induction of apoptosis, evidenced by an increased sub-diploid cell population, and G2/M phase cell-cycle arrest. Inhibition of tubulin polymerization. In vivo antitumor activity also reported in the Ehrlich ascites carcinoma (EAC) model.	[64]
A549	Carotol	52.34 µM (MTT, 24 h)	Selective cytotoxic activity.	[44]
A549	EO of *Pittosporum glabratum*	35.32 µg/mL (MTT, 48 h)	Selective cytotoxic activity. G1 cell-cycle arrest via downregulation of CDK2/cyclin E and CDK4/cyclin D3 and upregulation of p21. Induction of mitochondrial apoptosis (Bax/Bcl-2 modulation, Cyt c release, caspase-9 and caspase-3 activation, PARP cleavage). Inhibition of migration and invasion associated with downregulation of MMP-2, MMP-9, and N-cadherin.	[67]
A549	EO of *Origanum onites*	4.79 µg/mL (MTT, 24 h)	Selective cytotoxic activity. Induction of apoptosis (AO/EtBr staining) associated with increased ROS production. Inhibition of cell proliferation and migration.	[68]
A-549-C5	EO of *Thymus bovei* Benth.	8.62 µg/mL (MTT, 24 h)	Selective cytotoxic activity.	[59]
A549	Citronellol	69.10 µg/mL (MTT, 24 h)	Selective antiproliferative activity. Induction of necroptosis via TNF-α signaling and ROS accumulation. G1 phase cell-cycle arrest.	[65]
NCI-H1299	Citronellol	49.74 µg/mL (MTT, 24 h)	Selective antiproliferative activity. TNF-α-dependent necroptosis with RIP1/RIP3 upregulation, caspase-3/8 downregulation, and ROS accumulation. G1 cell-cycle arrest.	[65]
NCI-H460	6,7-dehydroroyleanone nanoencapsulated (in hybrid nanoparticles)	0.53 µg/mL (SRB, 72 h)	Selective cytotoxic activity, including drug-resistant NCI-H460/R cells. The nanoencapsulated formulation overcomes P-glycoprotein-mediated resistance, with cytotoxic activity independent of P-gp expression.	[66]
NCI-H460	Eugenol	N/A	Selective cytotoxic activity associated with inhibition of mitochondrial oxidative phosphorylation and increased ROS production.	[26]
NCI-H460	*Syzygium aromaticum* EO (formulated with Tween-20)	N/A	Selective cytotoxic activity associated with mitochondrial dysfunction, OXPHOS uncoupling, and oxidative stress induction.	[26]
NCI-H460	EO of *Zanthoxylum rhetsa* fruit	1.79 µL/mL (MTT, 24 h)	Selective cytotoxic activity. Induction of apoptosis.	[69]
NCI-H460	EO of *Alpinia nelumboides* rhizome	98.28 µg/mL (SRB, 48 h)	Selective cytotoxic activity.	[70]
H727	3-Carene	12.61 µg/mL (MTT, 48 h)	Selective antiproliferative activity. Induction of apoptosis associated with increased Bax and caspase-3 expression and G0/G1 cell-cycle arrest.	[25]

**Table 6:** In vivo studies with EOs and single compound formulations in lung cancer models.

Model	EO/Single Compound	Dosage	Effects	Reference
Athymic nude mice (BALB/c) with A549 xenografts	Carvacrol nanoemulsion	50 and 100 mg/kg, oral administration (three times per week for 4 weeks)	Dose-dependent tumor growth inhibition. Reduction in tumor weight (34.2–62.1%). Induction of apoptosis associated with mitochondrial and oxidative stress-related mechanisms. Improvement of body weight in treated animals.	[61]
Athymic nude mice (BALB/c) with A549 xenografts	EO of *Pittosporum glabratum*	100 mg/kg, intraperitoneal administration (daily for 15 days)	Dose-dependent reduction in tumor mass (up to ~56%) without overt systemic toxicity. No significant alterations in body weight, organ weights, or hematological parameters were observed.	[67]

**Table 7:** In vitro studies with EOs and their single compounds on colorectal cancer cell lines.

Cell Line	EO/Single Compound	IC_50_	Effects	Reference
HCT116	EO of *Thymus hirtus ssp. algeriensis*	N/A	Selective antiproliferative activity. Cell-cycle arrest associated with reduced cyclin D1 expression and p21/p53 upregulation. Induction of apoptosis via caspase-8, -9 and -3 activation and PARP cleavage. Upregulation of death receptors (DR4/DR5) and inhibition of pro-survival signaling pathways (STAT3/JAK2, AKT).	[72]
HCT116	EO of *Ocimum forskolei*	5.34 µg/mL (MTT, 72 h)	Selective cytotoxic activity. Inhibition of cell proliferation and clonogenic survival. Cell-cycle arrest associated with downregulation of cyclin D1 and PCNA. Induction of apoptosis with caspase-3 activation and Bcl-2 downregulation.	[71]
HCT 116 (p53 wt & null)	EO of *Calocedrus formosana* wood	N/A	Selective antiproliferative activity. ROS-associated induction of autophagy and apoptosis, evidenced by ULK1/Atg activation, LC3-II conversion, caspase-3/-9 activation, and PARP cleavage. Inhibition of SIRT1 activity leading to cell death independently of p53 status.	[74]
HCT116	Terpinen-4-ol	661 µM (WST-8, 24 h)	Selective cytotoxic activity. Induction of apoptotic cell death associated with mitochondrial ROS generation, caspase-3/7 activation, and Annexin V positivity, without LDH-associated necrosis.	[75]
HCT116	EO of *Juniperus communis*	40.51 µg/mL (MTT, 24 h)	Selective cytotoxic activity. Apoptosis associated with mitochondrial dysfunction, Bax/Bcl-2 modulation, caspase-3 activation, loss of mitochondrial membrane potential, and cytochrome-c release. Inhibition of AKT and ERK signaling and reduced clonogenic survival.	[73]
HCT116	Carotol	25.68 µM (MTT, 24 h)	Selective cytotoxic activity.	[44]
HCT116	EO of *Chrysopogon zizanioides*	62.95 µg/mL (Alamar Blue, 48 h)	Selective cytotoxic activity. Inhibition of cancer cell proliferation. In silico analyses suggest involvement of AKT1 and STAT3 pathways.	[82]
HCT116	EO of *Illicium verum*	50.34 µg/mL (MTT, 48 h)	Selective cytotoxic activity. Induction of apoptosis associated with mitochondrial membrane depolarization and chromatin condensation. Inhibition of cell migration, invasion, and colony formation.	[76]
HCT-15	EO of *Rosmarinus officinalis*	1.72 µg/mL (MTT, 72 h)	Selective cytotoxic activity.	[48]
HT-29	EO of *Cedrus atlantica*	N/A	Selective cytotoxic activity. Induction of apoptosis associated with mitochondrial dysfunction, caspase-3/7 activation, and suppression of mitochondrial respiration.	[27]
HT-29	*Syzygium aromaticum* EO nanoemulsion	74.8 µg/mL (MTT, 48 h)	Selective cytotoxic activity. Induction of apoptotic cell death evidenced by caspase-3 upregulation, increased Sub-G1 population, and AO/PI staining.	[77]
HT-29	3-Cyclohexene-1-methanol	0.76 µg/mL (MTT, 24 h)	Selective cytotoxic activity. Caspase-independent apoptosis associated with altered cell-cycle progression and activation of stress-related MAPK pathways (p53-independent response).	[81]
RKO	EO of *Otanthus maritimus*	0.34 µL/mL (MTS, 72 h)	Selective antiproliferative activity	[36]
RKO	EO of *Artemisia campestris* subsp. *maritima*	0.35 µL/mL (MTS, 72 h)	Selective antiproliferative activity	[36]
RKO	EO of *Seseli tortuosum*	0.034 µL/mL (MTS, 72 h)	Selective antiproliferative activity	[36]
RKO	EO of *Eryngium maritimum*	0.47 µL/mL (MTS, 72 h)	Selective antiproliferative activity	[36]
RKO	Terpinen-4-ol	381 µM (WST-8, 24 h)	Selective antiproliferative activity	[75]
Caco-2	Eugenol	458 µM (MTT, 24 h)	Selective cytotoxic activity. Induction of cell-cycle perturbation and apoptotic/necrotic cell death after prolonged exposure.	[80]
Caco-2	Cinnamaldehyde	166 µM (MTT, 24 h)	Selective antiproliferative activity associated with cell-cycle modulation and induction of apoptotic/necrotic cell death.	[80]
Caco-2	EO of *Foeniculum vulgare*	300 µg/mL (NRU, 24 h)	Selective cytotoxic activity. Induction of apoptosis and cell-cycle arrest with reduced proliferation.	[79]
Caco-2	EO of *Syzygium aromaticum*	150 µg/mL (NRU, 24 h)	Selective cytotoxic activity associated with apoptosis induction and inhibition of proliferation.	[79]
Caco-2	*Foeniculum vulgare* + *Syzygium aromaticum* EO mixture (2:1, v/v)	73 µg/mL (NRU, 24 h)	Selective cytotoxic activity with synergistic interaction (CI < 1). Induction of apoptosis and S- and G2/M-phase cell-cycle arrest, associated with reduced Bcl-2 and Ki-67 expression, oxidative stress induction, and extensive DNA fragmentation.	[79]
Caco-2	Eugenol	N/A	Selective antiproliferative activity with reduced cell viability and clonogenic growth, associated with mitochondrial stress and activation of apoptotic signaling.	[78]
Caco-2	Cinnamaldehyde	N/A	Selective antiproliferative activity with marked inhibition of proliferation and clonogenicity, associated with modulation of inflammatory and metabolic stress pathways.	[78]
SW-620	Eugenol	918 µM (MTT, 24 h)	Selective cytotoxic activity in metastatic colorectal cancer cells with predominant necrotic cell death at effective concentrations.	[80]
SW-620	Cinnamaldehyde	62 µM (MTT, 24 h)	Selective cytotoxic activity in metastatic colorectal cancer cells, with cell cycle arrest and prevalent necrotic cell death.	[80]
SW-620	Eugenol	N/A	Selective antiproliferative activity with reduced cell viability and clonogenic growth, associated with mitochondrial stress and apoptotic signaling activation.	[78]
SW-620	Cinnamaldehyde	N/A	Selective antiproliferative activity with strong suppression of clonogenic growth and modulation of cytokine-related and metabolic stress pathways.	[78]
LS-174-D3	3-Cyclohexene-1-methanol	0.6 µg/mL (MTT, 24 h)	Selective cytotoxic activity associated with caspase-dependent apoptosis and G2/M cell-cycle arrest, accompanied by inhibition of AKT and ERK1/2 signaling (p53-dependent response)	[81]
LS-174-D3	EO of *Thymus bovei* Benth.	9.30 µg/mL (MTT, 24 h)	Selective cytotoxic activity.	[59]

**Table 8:** In vivo studies with EOs and single compounds formulations in colorectal cancer models.

Model	EO/Single Compound	Dosage	Effects	Reference
HCT116 colorectal cancer xenograft (ICR-SCID mice)	Terpinen-4-ol	200 mg/kg, subcutaneous injection (every 3 days, 5 doses)	Significant inhibition of tumor growth. Increased apoptotic features in tumor tissue (cleaved caspase-3) and oxidative stress markers (8-OHdG). No significant changes in body weight or serum toxicity markers (ALT, creatinine).	[75]
ApcMin/+ mice (C57BL/6J-ApcMin/+)	Pogostone	40 mg/kg/day, oral administration (6 weeks)	Significant reduction in intestinal polyp number and size without overt systemic toxicity. Ex vivo analyses of intestinal tissues indicated improved epithelial barrier integrity and modulation of immune responses and gut microbiota composition, supporting a chemopreventive effect.	[83]
Swiss albino mice (LPS-induced colon carcinogenesis)	EO of *Thymus hirtus* ssp. *algeriensis*	12.5 and 50 mg/mL, oral administration (7 days)	Inhibition of LPS-induced colon carcinogenesis, with preservation of colon length and reduced histopathological damage, including decreased tumor budding. No overt systemic toxicity was observed. Ex vivo analyses of colon tissues indicated suppression of pro-tumorigenic and pro-inflammatory markers (including VEGF and anti-apoptotic proteins) and modulation of apoptosis- and inflammation-related signaling pathways, including death receptor-mediated apoptosis, supporting the observed in vivo chemopreventive effects.	[72]

**Table 9:** In vitro studies with EOs and single compounds on hepatocellular cancer cell lines.

Cell Line	EO/Single Compound	IC_50_	Effects	Reference
Hep3B	Curcumol	N/A	Selective antiproliferative activity. Dual inhibition of STAT3 and HIF-1α signaling, with suppression of p-STAT3 (via JAK1/2/Src) and reduced HIF-1α synthesis (via mTOR/p70S6K/eIF4E and MAPK), associated with PD-L1 downregulation and reduced tumor cell proliferation and angiogenic potential.	[90]
HepG2	EO of *Origanum vulgare*	236 µg/mL (MTT, 24 h)	Selective cytotoxic activity with cancer-specific reduction in viability and associated morphological alterations.	[84]
HepG2	Thymol	289.5 µg/mL (MTT, 24 h)	Selective cytotoxic activity with cancer-specific reduction in viability and associated morphological alterations.	[84]
HepG2	Carvacrol	48.3 µg/mL (MTT, 24 h)	Selective cytotoxic activity with cancer-specific reduction in viability and associated morphological alterations.	[84]
HepG2	Combined EOs of *Curcuma longa* and *Nigella sativa*	10.16 µg/100 µL (MTT, 48 h)	Selective cytotoxic activity, with potency comparable to 5-fluorouracil. Reduced cancer cell proliferation and protein denaturation. In silico analyses identified NOX2, NF-κB, and MDM2 as potential molecular targets. The combined EOs exhibited higher cytotoxic potency than the individual oils and their major isolated constituents.	[89]
HepG2	EO of *Croton matourensis*	28.5 µg/mL (Alamar Blue, 72 h)	Selective cytotoxic activity. Induction of apoptosis evidenced by phosphatidylserine externalization and internucleosomal DNA fragmentation.	[87]
HepG2	EO of *Anisosciadium lanatum* Boiss.	11.3 µg/mL (MTT, 24 h)	Selective cytotoxic activity. Modulation of apoptotic markers, including downregulation of Bcl-2 and NF-κB and upregulation of caspase-3 and CYP-1A1. In silico docking suggested potential interactions of major constituents with Bcl-2 and caspase-3.	[86]
HepG2	EO of *Chamaecyparis lawsoniana*	15.34 µg/mL (MTT, 24 h)	Selective cytotoxic activity. Exploratory in silico docking suggested potential interactions of major constituents (e.g., cis-abienol, trans-ferruginol) with protein targets implicated in proliferation and apoptosis, including EGFR, Mcl-1, and caspase-8.	[85]
HepG2	*Origanum glandulosum* EO nanoencapsulated (alginate nanocapsules)	54.93 µg/mL (MTT, 48 h)	Selective cytotoxic activity. (Nanoencapsulation enhanced cytotoxic potency compared with the free EO, potentially preserving the activity of major phenolic constituents such as thymol and carvacrol.)	[88]
HepG2	Carotol	38.21 µM (MTT, 24 h)	Selective cytotoxic activity.	[44]
HepG2	EO of *Rosmarinus officinalis*	21.91 µg/mL (MTT, 72 h)	Selective cytotoxic activity.	[48]
HepG2	EO of *Alpinia nelumboides* rhizome	189.87 µg/mL (SRB, 48 h)	Selective cytotoxic activity.	[70]
HepG2	EO of *Euphorbia esula*	50.67 µg/mL (MTT, 24 h)	Selective cytotoxic activity. Induction of mitochondrial apoptosis (Bax/Bcl-2 modulation, caspase activation) associated with increased ROS levels.	[91]
HepG2	EO of *Nigella sativa* nanoemulsion	55.7 µg/mL (MTT, 24 h)	Selective cytotoxic activity. Induction of apoptosis associated with Bax upregulation and Bcl-2 downregulation.	[92]
Huh-7	EO of Nigella sativa nanoemulsion	35.5 µg/mL (MTT, 24 h)	Selective cytotoxic activity. Induction of apoptosis associated with Bax upregulation and Bcl-2 downregulation.	[92]

**Table 10:** In vivo studies with EOs and single compound formulations in hepatocellular cancer models.

Model	EO/Single Compound	Dosage	Effects	Reference
Athymic nude mice (BALB/c) with Hep3B xenografts	Curcumol	3 and 30 mg/kg, oral administration (three times per week for 30 days)	Dose-dependent inhibition of tumor growth without evidence of systemic toxicity. Reduced tumor volume and weight were accompanied by decreased expression of p-STAT3, HIF-1α, PD-L1, and VEGF in tumor tissues, as assessed ex vivo by Western blotting and immunohistochemistry.	[90]
C.B-17 SCID mice with HepG2 xenografts	EO of *Croton matourensis*	40 and 80 mg/kg/day, intraperitoneal administration (once daily for 21 consecutive days)	Dose-dependent reduction in tumor mass (up to ~56%) without overt systemic toxicity. No significant alterations in body weight, organ weights, or hematological parameters were observed.	[87]

**Table 11:** In vitro studies with EOs and single compounds on oral and hypopharyngeal cancer cell lines.

Model	EO/Single Compound	IC_50_	Effects	Reference
FaDu (hypopharyngeal squamous cell carcinoma)	Carvacrol	9.61 µM (MTT, 24 h)	Selective antiproliferative activity. Cell-cycle arrest and apoptosis associated with inhibition of ornithine decarboxylase (ODC) and hyaluronidase (HYAL) activities.	[93]
KB (oral squamous carcinoma)	EO of *Thymus caramanicus*	0.44 µL/mL (MTT, 24 h)	Selective cytotoxic activity.	[94]
KON (oral squamous carcinoma)	*Psidium guajava* leaf EO nanoemulsions	62.49 mg/mL (MTT, 24 h)	Selective cytotoxic activity. Induction of apoptosis. Inhibition of colony formation and cell invasion.	[95]

**Table 12:** In vitro studies with EOs and single compounds on cervical cancer cell lines.

Cell Line	EO/Single Compound	IC_50_	Effects	Reference
HeLa	EO of *Erigeron canadensis* L.	18.06 µg/mL (MTT, 24 h)	Selective cytotoxic activity. G2/M cell-cycle arrest and caspase-dependent apoptosis associated with oxidative stress modulation, mitochondrial dysfunction, loss of mitochondrial membrane potential, and activation of caspase-3/-9/-12. EO more potent than limonene alone, indicating contribution of minor constituents (e.g., nerolidol, α-pinene).	[99]
HeLa	Combined EOs of *Curcuma longa* and *Nigella sativa*	148.3 µg/mL (MTT, 48 h)	Selective cytotoxic activity. Inhibition of cancer cell proliferation and protein denaturation. In silico analyses identified NOX2, NF-κB, and MDM2 as potential molecular targets. Combined EOs showed higher potency than individual oils and major isolated constituents.	[89]
HeLa	EO of *Orthosiphon schimperi* Benth.	29.44 µg/mL (MTT, 24 h)	Selective cytotoxic activity. Induction of apoptosis evidenced by PARP-1 cleavage.	[97]
HeLa	EO of *Myrtus communis*	8.12 µg/mL (MTT, 72 h)	Selective cytotoxic activity.	[49]
HeLa-R2	EO of *Thymus bovei* Benth.	7.22 µg/mL (MTT, 24 h)	Selective cytotoxicity.	[59]
Hep-2	EO of *Piper eriopodon*	33 µg/mL (MTT, 24 h)	Selective cytotoxic activity.	[98]

**Table 13:** In vitro studies with EOs and single compounds on hematological cancer cell lines.

Cell Line	EO/Single Compound	IC_50_	Effects	Reference
HL-60	EO of *Baccharis milleflora* (Less.)	23.06 µg/mL (MTT, 24 h)	Selective cytotoxic activity. Induction of apoptotic and necrotic cell death.	[100]
HL-60	EO of *Glandora rosmarinifolia*	35.75 µg/mL (MTS, 72 h)	Selective cytotoxic activity. Induction of apoptosis associated with inhibition of topoisomerase II activity and G0/G1 cell-cycle arrest.	[101]
HL-60	EO of *Ptychotis verticillata*	N/A	Selective cytotoxic activity. Induction of apoptosis associated with oxidative stress.	[102]
HL-60	Carvacrol + Thymol	N/A	Selective cytotoxic activity. ROS-mediated, caspase-dependent apoptosis.	[102]
HL-60R	EO of *Glandora rosmarinifolia*	37.0 µg/mL (MTS, 72 h)	Selective cytotoxic activity. Induction of apoptosis associated with inhibition of topoisomerase II activity and G0/G1 cell-cycle arrest, confirming activity also in drug-resistant cells.	[101]
HL-60R	EO of *Ptychotis verticillata*	N/A	Selective cytotoxic activity. Induction of apoptosis associated with oxidative stress, confirming activity in drug-resistant cells.	[102]
HL-60R	Carvacrol + Thymol	N/A	Selective cytotoxic activity. ROS-mediated apoptosis, confirming activity in drug-resistant cells.	[102]
Jurkat	EO of *Baccharis milleflora* (Less.)	42.91 µg/mL (MTT, 24 h)	Selective cytotoxic activity. Induction of apoptotic and necrotic cell death.	[100]
Raji	EO of *Baccharis milleflora* (Less.)	39.15 µg/mL (MTT, 24 h)	Selective cytotoxic activity. Induction of apoptosis associated with G0/G1 cell-cycle arrest and reduction in S and G2/M phases.	[100]
Raji	EO of *Myrtus communis*	27.32 µg/mL (MTT, 24 h)	Selective antiproliferative activity.	[49]
K562	EO of *Alpinia galanga*	41.55 µg/mL (MTT, 48 h)	Selective cytotoxic activity. Induction of apoptosis.	[104]
K562	EO of *Kelussia odoratissima*	70.0 µg/mL (MTT, 48 h)	Selective cytotoxic effect	[103]
K562	EO of *Ptychotis verticillata*	N/A	Selective cytotoxic activity. Cell death associated with oxidative stress.	[102]
K562	Carvacrol + Thymol	N/A	Selective cytotoxic activity. ROS-mediated cell death.	[102]
K562	EO of *Varthemia iphionoides*	87.88 µg/mL (MTT, 24 h)	Selective cytotoxic activity. Induction of apoptosis.	[50]
K562	EO of *Nectandra lanceolata* stem bark	N/A	Selective antiproliferative activity; TGI = 14.6 ± 2.1 µg/mL in K562 cells.	[105]
KG-1	EO of *Ptychotis verticillata*	N/A	Selective cytotoxic activity. Cell death associated with oxidative stress.	[102]
KG-1	Carvacrol + Thymol	N/A	Selective cytotoxic activity. ROS-mediated cell death.	[102]
MOLT-3	6,7-Dehydroroyleanone	N/A	Selective cytotoxic activity. Caspase-dependent apoptosis via the intrinsic pathway, characterized by marked sub-G1 accumulation and depletion of cells across all cell-cycle phases.	[66]

**Table 14:** In vitro studies with EOs and single compounds on prostate cancer cell lines.

Cell Line	EO/Single Compound	IC_50_	Effects	Reference
DU145	EO of *Pinus mugo*	70.3 µg/mL (MTT, 24 h)	Selective cytotoxic activity. Induction of apoptosis associated with oxidative stress and impairment of STAT3 signaling.	[107]
PC-3	EO of *Varthemia iphionoides*	145.3 µg/mL (MTT, 24 h)	Selective cytotoxic activity.	[50]
PC-3	EO of *Hedychium spicatum*	21.88 µg/mL (MTT, 24 h)	Selective cytotoxic activity. Induction of intrinsic mitochondrial apoptosis associated with ROS accumulation, loss of mitochondrial membrane potential, activation of caspase-3, -8, and -9, and modulation of Bcl-2 family proteins, including decreased Bcl-2 expression and increased Bax and Bad levels.	[108]

**Table 15:** In vitro studies with EOs and single compounds on ovarian cancer cell lines.

Cell Line	EO/Single Compound	IC_50_	Effects	Reference
PA-1	β-Caryophyllene	11.46 µM (MTT, 24 h)	Selective antiproliferative activity. Induction of apoptosis associated with caspase-3 activation and PARP cleavage, together with S-phase cell-cycle arrest.	[109]
OAW 42	β-Caryophyllene	11.88 µM (MTT, 24 h)	Selective antiproliferative activity. Induction of apoptosis associated with caspase-3 activation and PARP cleavage, together with S-phase cell-cycle arrest.	[109]
SKOV3	EO of *Kelussia odoratissima*	120 µg/mL (MTT, 24 h)	Selective cytotoxic activity.	[103]

**Table 16:** In vitro studies with EOs and single compounds on Brain Cancer cell lines.

Cell Line	EO/Single Compound	IC_50_	Effects	Reference
T98 (glioblastoma)	Isofuranodiene	126.6 µM (SRB, 48 h)	Selective cytotoxic activity. G1-phase cell-cycle arrest and ROS-mediated necrotic cell death.	[110]
U251 (glioblastoma)	Isofuranodiene	245.5 µM (SRB, 48 h)	Selective cytotoxic activity. G1-phase cell-cycle arrest and ROS-mediated necrotic cell death.	[110]
U87 (glioblastoma)	Isofuranodiene	158.1 µM (SRB, 48 h)	Selective cytotoxic activity. G1-phase cell-cycle arrest and ROS-mediated necrotic cell death.	[110]
U87 (glioblastoma)	Eugenol + β-caryophyllene	N/A	Selective cytotoxic activity. Reduced proliferation and migration, G0/G1 cell-cycle arrest, and transcriptional modulation of cell-cycle- and invasion-related genes. Synergistic activity observed for the combined treatment.	[111]
SH-SY5Y (neuroblastoma)	Myrcene	N/A	Selective cytotoxic activity. Induction of apoptosis associated with oxidative stress and mitochondrial dysfunction.	[112]

## Data Availability

No new data were created or analyzed in this study. Data sharing is not applicable to this article.

## Supplementary Materials

The following supporting information can be downloaded at: https://www.mdpi.com/article/10.3390/ijms27104379/s1.

## References

[1] De Sousa D.P., Damasceno R.O.S., Amorati R., Elshabrawy H.A., De Castro R.D., Bezerra D.P., Nunes V.R.V., Gomes R.C., Lima T.C. (2023). Essential Oils: Chemistry and Pharmacological Activities. Biomolecules.

[2] (2021). Aromatic Natural Raw Materials—Vocabulary.

[3] Caneschi A., Bardhi A., Barbarossa A., Zaghini A. (2023). Plant Essential Oils as a Tool in the Control of Bovine Mastitis: An Update. Molecules.

[4] Burčul F., Blažević I., Radan M., Politeo O. (2020). Terpenes, Phenylpropanoids, Sulfur and Other Essential Oil Constituents as Inhibitors of Cholinesterases. CMC.

[5] Masyita A., Mustika Sari R., Dwi Astuti A., Yasir B., Rahma Rumata N., Emran T.B., Nainu F., Simal-Gandara J. (2022). Terpenes and Terpenoids as Main Bioactive Compounds of Essential Oils, Their Roles in Human Health and Potential Application as Natural Food Preservatives. Food Chem. X.

[6] Breitmaier E. (2006). Terpenes: Flavors, Fragrances, Pharmaca, Pheromones.

[7] Hosseini M., Pereira D.M. (2023). The Chemical Space of Terpenes: Insights from Data Science and AI. Pharmaceuticals.

[8] Bhavaniramya S., Vishnupriya S., Al-Aboody M.S., Vijayakumar R., Baskaran D. (2019). Role of Essential Oils in Food Safety: Antimicrobial and Antioxidant Applications. Grain Oil Sci. Technol..

[9] Perveen S. (2021). Introductory Chapter: Terpenes and Terpenoids. Terpenes and Terpenoids—Recent Advances.

[10] Hyldgaard M., Mygind T., Meyer R.L. (2012). Essential Oils in Food Preservation: Mode of Action, Synergies, and Interactions with Food Matrix Components. Front. Microbiol..

[11] Alves-Silva J.M., Zuzarte M., Marques C., Girão H., Salgueiro L. (2019). Protective Effects of Phenylpropanoids and Phenylpropanoid-Rich Essential Oils on the Cardiovascular System. MRMC.

[12] Ferrer J.-L., Austin M.B., Stewart C., Noel J.P. (2008). Structure and Function of Enzymes Involved in the Biosynthesis of Phenylpropanoids. Plant Physiol. Biochem..

[13] Seigler D.S. (1998). Plant Secondary Metabolism.

[14] Bakkali F., Averbeck S., Averbeck D., Idaomar M. (2008). Biological Effects of Essential Oils—A Review. Food Chem. Toxicol..

[15] Ivanova S., Gvozdeva Y., Staynova R., Grekova-Kafalova D., Nalbantova V., Benbassat N., Koleva N., Ivanov K. (2025). Essential Oils—A Review of the Natural Evolution of Applications and Some Future Perspectives. Pharmacia.

[16] Mutlu-Ingok A., Devecioglu D., Dikmetas D.N., Karbancioglu-Guler F., Capanoglu E. (2020). Antibacterial, Antifungal, Antimycotoxigenic, and Antioxidant Activities of Essential Oils: An Updated Review. Molecules.

[17] Spisni E., Petrocelli G., Imbesi V., Spigarelli R., Azzinnari D., Donati Sarti M., Campieri M., Valerii M.C. (2020). Antioxidant, Anti-Inflammatory, and Microbial-Modulating Activities of Essential Oils: Implications in Colonic Pathophysiology. Int. J. Mol. Sci..

[18] Mohamed Abdoul-Latif F., Ainane A., Houmed Aboubaker I., Mohamed J., Ainane T. (2023). Exploring the Potent Anticancer Activity of Essential Oils and Their Bioactive Compounds: Mechanisms and Prospects for Future Cancer Therapy. Pharmaceuticals.

[19] Garzoli S., Alarcón-Zapata P., Seitimova G., Alarcón-Zapata B., Martorell M., Sharopov F., Fokou P.V.T., Dize D., Yamthe L.R.T., Les F. (2022). Natural Essential Oils as a New Therapeutic Tool in Colorectal Cancer. Cancer Cell Int..

[20] Page M.J., McKenzie J.E., Bossuyt P.M., Boutron I., Hoffmann T.C., Mulrow C.D., Shamseer L., Tetzlaff J.M., Akl E.A., Brennan S.E. (2021). The PRISMA 2020 Statement: An Updated Guideline for Reporting Systematic Reviews. BMJ.

[21] Dinç H., Yiğin A., Koyuncu İ., Aslan M. (2022). Investigation of the Anticancer and Apoptotic Effect of *Micromeria congesta* under In Vitro Conditions and Detection of Related Genes by Real-Time PCR. Vet. Res. Forum.

[22] Askari N., Parvizpour S., Marashi S.M.B., Baghery F., Khanamani Falahati-pour S. (2023). In Vitro and Pharmacoinformatics-Based Phytochemical Screening for Anticancer Impacts of Pistachio Hull Essential Oil on AGS, PLC/PRF/5, and CACO2 Cell Lines. Mol. Biol. Rep..

[23] Sogutlu F., Pekerbas M., Boztas G., Bayram E., Pariltay E., Cogulu O., Biray Avci C. (2025). The Anticancer Potential of *Origanum onites* L. in Gastric Cancer through Epigenetic Alterations. BMC Complement. Med. Ther..

[24] Günes-Bayir A., Kocyigit A., Güler E.M., Bilgin M.G., Ergün İ.S., Dadak A. (2018). Effects of Carvacrol on Human Fibroblast (WS-1) and Gastric Adenocarcinoma (AGS) Cells In Vitro and on Wistar Rats In Vivo. Mol. Cell Biochem..

[25] Balusamy S.R., Samad A., Singh P., Sunderraj S., Elsadek M.F., Altwaijry N., Sukweenadhi J., Perumalsamy H. (2026). Comparative Anti-Cancer Properties of Carene Isoforms Induced Apoptotic Cell Death in Stomach and Lung Cancer Cell Lines. Naunyn-Schmiedeberg’s Arch. Pharmacol..

[26] Munteanu A., Gogulescu A., Șoica C., Mioc A., Mioc M., Milan A., Lukinich-Gruia A.T., Pricop M.-A., Jianu C., Banciu C. (2024). In Vitro and In Silico Evaluation of Syzygium Aromaticum Essential Oil: Effects on Mitochondrial Function and Cytotoxic Potential Against Cancer Cells. Plants.

[27] Gruin S., Crețu O., Mioc A., Mioc M., Prodea A., Atyim E., Lukinich-Gruia A.T., Pricop M.-A., Gogulescu A., Șoica C. (2025). Evaluation of *Cedrus atlantica* Essential Oil: Chemical Composition, Anticancer Activity and Molecular Docking Studies. Molecules.

[28] Maksimović T., Minda D., Șoica C., Mioc A., Mioc M., Colibășanu D., Lukinich-Gruia A.T., Pricop M.-A., Jianu C., Gogulescu A. (2025). Anticancer Potential of *Cymbopogon citratus* L. Essential Oil: In Vitro and In Silico Insights into Mitochondrial Dysfunction and Cytotoxicity in Cancer Cells. Plants.

[29] Othman R., Moarbes V., Zaatar M.T., Antonios D., Roufayel R., Beyrouthy M., Fajloun Z., Sabatier J.-M., Karam M. (2025). Cytotoxic Activity of Essential Oils from Middle Eastern Medicinal Plants on Malignant Keratinocytes. Molecules.

[30] Milosevic Markovic M., Anicic B., Lazarevic M., Jaksic Karisik M., Mitic D., Milovanovic B., Ivanovic S., Pecinar I., Petrovic M., Petrovic M. (2025). Cytotoxic Effects of *Thymus serpyllum* L. and *Mentha* × *piperita* L. Essential Oils on Basal Cell Carcinoma—An In Vitro Study. Life.

[31] Mothana R.A., Nasr F.A., Khaled J.M., AL-Zharani M., Noman O.M., Abutaha N., Al-Rehaily A.J., Almarfadi O.M., Kumar A., Kurkcuoglu M. (2019). Analysis of Chemical Composition and Assessment of Cytotoxic, Antimicrobial, and Antioxidant Activities of the Essential Oil of *Meriandra dianthera* Growing in Saudi Arabia. Molecules.

[32] Nasr F.A., Noman O.M., Alqahtani A.S., Qamar W., Ahamad S.R., Al-Mishari A.A., Alyhya N., Farooq M. (2020). Phytochemical Constituents and Anticancer Activities of *Tarchonanthus camphoratus* Essential Oils Grown in Saudi Arabia. Saudi Pharm. J..

[33] Saleh A.M., Al-Qudah M.A., Nasr A., Rizvi S.A., Borai A., Daghistani M. (2017). Comprehensive Analysis of the Chemical Composition and In Vitro Cytotoxic Mechanisms of *Pallines Spinosa* Flower and Leaf Essential Oils Against Breast Cancer Cells. Cell Physiol. Biochem..

[34] Azani H., Homayouni Tabrizi M., Neamati A., Khadem F., Khatamian N. (2022). The *Ferula Assa-foetida* Essential Oil Nanoemulsion (FAEO-NE) as the Selective, Apoptotic, and Anti-Angiogenic Anticancer Compound in Human MCF-7 Breast Cancer Cells and Murine Mammary Tumor Models. Nutr. Cancer.

[35] Salehi F., Jamali T., Kavoosi G., Ardestani S.K., Vahdati S.N. (2020). Stabilization of Zataria Essential Oil with Pectin-Based Nanoemulsion for Enhanced Cytotoxicity in Monolayer and Spheroid Drug-Resistant Breast Cancer Cell Cultures and Deciphering Its Binding Mode with gDNA. Int. J. Biol. Macromol..

[36] Beeby E., Magalhães M., Poças J., Collins T., Lemos M.F.L., Barros L., Ferreira I.C.F.R., Cabral C., Pires I.M. (2020). Secondary Metabolites (Essential Oils) from Sand-Dune Plants Induce Cytotoxic Effects in Cancer Cells. J. Ethnopharmacol..

[37] Benedetti S., Nasoni M.G., Luchetti F., Palma F. (2023). New Insights into the Cytotoxic Effects of *Thymus vulgaris* Essential Oil on the Human Triple-Negative Breast Cancer Cell Line MDA-MB-231. Toxicol. Vitr..

[38] Estanislao Gómez C.C., Aquino Carreño A., Pérez Ishiwara D.G., San Martín Martínez E., Morales López J., Pérez Hernández N., Gómez García M.C. (2016). *Decatropis bicolor* (Zucc.) Radlk Essential Oil Induces Apoptosis of the MDA-MB-231 Breast Cancer Cell Line. BMC Complement. Altern. Med..

[39] Bashlouei S.G., Karimi E., Zareian M., Oskoueian E., Shakeri M. (2022). *Heracleum persicum* Essential Oil Nanoemulsion: A Nanocarrier System for the Delivery of Promising Anticancer and Antioxidant Bioactive Agents. Antioxidants.

[40] Kadyrbay A., Ibragimova L.N., Iwan M., Ludwiczuk A., Biernasiuk A., Sakipova Z.B., Świątek Ł., Salwa K., Korga-Plewko A., Zhaparkulova K.A. (2025). Essential Oil from the Aerial Parts of *Artemisia serotina* Bunge (Winter Wormwood) Growing in Kazakhstan—Phytochemical Profile and Bioactivity. Molecules.

[41] Jamali T., Kavoosi G., Ardestani S.K. (2020). In-Vitro and in-Vivo Anti-Breast Cancer Activity of OEO (Oliveria Decumbens Vent Essential Oil) through Promoting the Apoptosis and Immunomodulatory Effects. J. Ethnopharmacol..

[42] Reyes-Vidal I., Tepale-Ledo I., Rivera G., Ortiz-Islas E., Pérez-Mora S., Pérez-Ishiwara D.G., Flores-Martinez Y.M., Lara-Rodríguez M., Gómez-García M.D.C. (2025). In Silico and In Vitro Evaluation of δ-Cadinene from *Decatropis bicolor* as a Selective Inhibitor of Human Cell Adhesion and Invasion Proteins. Cancers.

[43] Saini D., Chaudhary P.K., Chaudhary J.K., Kaur H., Verma G.K., Pramanik S.D., Roy P., Mirza-Shariff A.A., Prasad R. (2025). Molecular Mechanisms of Antiproliferative and Pro-Apoptotic Effects of Essential Oil Active Constituents in MCF7 and T24 Cancer Cell Lines: In Vitro Insights and in Silico Modelling of Proapoptotic Gene Product-Compound Interactions. Apoptosis.

[44] Sary H.G., Khedr M.A., Radwan M.M., Vinodh M., Orabi K.Y. (2025). Microbial Biotransformation of the Sesquiterpene Carotol: Generation of Hydroxylated Metabolites with Potential Cytotoxic and Target-Specific Binding Activities. Biomolecules.

[45] Sufina Nazar S., Ayyappan J.P. (2024). Mechanistic Evaluation of Myristicin on Apoptosis and Cell Cycle Regulation in Breast Cancer Cells. J. Biochem. Mol. Toxicol..

[46] Mandal A.K., Paudel S., Pandey A., Yadav P., Pathak P., Grishina M., Jaremko M., Emwas A.-H., Khalilullah H., Verma A. (2022). Guava Leaf Essential Oil as a Potent Antioxidant and Anticancer Agent: Validated through Experimental and Computational Study. Antioxidants.

[47] Uchôa A.F.C., Formiga A.L.D., Cardoso A.L.M.R., Pereira G.M.A., Carvalho L.M.M., Souza P.H.O., Silva A.L., Souza R.R.M., Sobral M.V., Silva M.S. (2025). Optimized and Functionalized Carvacrol-Loaded Nanostructured Lipid Carriers for Enhanced Cytotoxicity in Breast Cancer Cells. Pharmaceutics.

[48] Assaggaf H., Hachlafi N.E., Mrabti N.N., Taibi M., Elbouzidi A., Qasem A., Attar A., Alshabrmi F.M., Ming L.C., Moshawih S. (2025). *Rosmarinus officinalis* Suppresses Cancer Cell Viability and Inflammatory Mediators in RAW 264.7 Cells: In Vitro and in Silico Analysis. Discov. Oncol..

[49] Bayoudh A., Tarhouni N., Ben Mansour R., Mekrazi S., Sadraoui R., Kriaa K., Ahmed Z., Soussi A., Kallel I., Hadrich B. (2025). Integrated Network Pharmacology and Molecular Dynamics Reveal Multi-Target Anticancer Mechanisms of *Myrtus communis* Essential Oils. Pharmaceuticals.

[50] Abbas M., Abbas M., Kandil Y. (2019). Cytotoxic ACTIVITY of *Varthemia iphionoides* essential oil against various human cancer cell lines. Acta Pol. Pharm.-Drug Res..

[51] Nayila I., Sharif S., Lodhi M.S., Rehman M.F.U., Aman F. (2023). Synthesis, Characterization and Anti-Breast Cancer Potential of an Incensole Acetate Nanoemulsion from *Catharanthus roseus* Essential Oil; In Silico, In Vitro, and In Vivo Study. RSC Adv..

[52] Kurt M., Korcan S.E., Azarkan S.Y. (2026). Antimicrobial and Anticancer Evaluation of Juniperus Chinensis Seed Oil with Molecular Docking of α-Cedrol–AKT1. Acta Chim. Slov..

[53] Nayila I., Sharif S., Shahzad Lodhi M., Ullah R., Alotaibi A., Maqbool T., Hameed S. (2024). Formulation, Characterization and Evaluation of Anti-Breast Cancer Activity of 2-Carene Nanoemulsion; in Silico, in Vitro and In Vivo Study. Arab. J. Chem..

[54] AlNaimat S., Abu-Odeh A., Talib W.H. (2024). Anticancer and Antioxidant Activities of Essential Oils of *Chiliadenus iphionoides* from Jordan: In Vitro and In Vivo Study. Pharmacia.

[55] Rojas-Armas J.P., Arroyo-Acevedo J.L., Palomino-Pacheco M., Ortiz-Sánchez J.M., Calva J., Justil-Guerrero H.J., Castro-Luna A., Ramos-Cevallos N., Cieza-Macedo E.C., Herrera-Calderon O. (2022). Phytochemical Constituents and Ameliorative Effect of the Essential Oil from *Annona muricata* L. Leaves in a Murine Model of Breast Cancer. Molecules.

[56] Xu X., Li Q., Dong W., Zhao G., Lu Y., Huang X., Liang X. (2023). *Cinnamon cassia* Oil Chitosan Nanoparticles: Physicochemical Properties and Anti-Breast Cancer Activity. Int. J. Biol. Macromol..

[57] Mohamed N., Ismail H., Nasr G.M., Abdel-Ghany S., Arneth B., Sabit H. (2025). Anti-Tumor Potential of Frankincense Essential Oil and Its Nano-Formulation in Breast Cancer: An In Vivo and In Vitro Study. Pharmaceutics.

[58] Fazelifar P., Tabrizi M.H., Rafiee A. (2021). The *Arachis hypogaea* Essential Oil Nanoemulsion as an Efficient Safe Apoptosis Inducer in Human Lung Cancer Cells (A549). Nutr. Cancer.

[59] Hassan S., Berchová-Bímová K., Šudomová M., Malaník M., Šmejkal K., Rengasamy K. (2018). In Vitro Study of Multi-Therapeutic Properties of *Thymus bovei* Benth. Essential Oil and Its Main Component for Promoting Their Use in Clinical Practice. J. Clin. Med..

[60] Jamali T., Kavoosi G., Jamali Y., Mortezazadeh S., Ardestani S.K. (2021). In-Vitro, in-Vivo, and in-Silico Assessment of Radical Scavenging and Cytotoxic Activities of *Oliveria decumbens* Essential Oil and Its Main Components. Sci. Rep..

[61] Khan I., Bahuguna A., Kumar P., Bajpai V.K., Kang S.C. (2018). In Vitro and In Vivo Antitumor Potential of Carvacrol Nanoemulsion against Human Lung Adenocarcinoma A549 Cells via Mitochondrial Mediated Apoptosis. Sci. Rep..

[62] Niu Q., Sun H., Liu C., Li J., Liang C., Zhang R., Ge F., Liu W. (2020). Croton Tiglium Essential Oil Compounds Have Anti-Proliferative and pro-Apoptotic Effects in A549 Lung Cancer Cell Lines. PLoS ONE.

[63] Yousefian Rad E., Homayouni Tabrizi M., Ardalan P., Seyedi S.M.R., Yadamani S., Zamani-Esmati P., Haghani Sereshkeh N. (2020). *Citrus lemon* Essential Oil Nanoemulsion (CLEO-NE), a Safe Cell-Depended Apoptosis Inducer in Human A549 Lung Cancer Cells with Anti-Angiogenic Activity. J. Microencapsul..

[64] Fatima K., Wani Z.A., Meena A., Luqman S. (2021). Geraniol Exerts Its Antiproliferative Action by Modulating Molecular Targets in Lung and Skin Carcinoma Cells. Phytother. Res..

[65] Yu W.-N., Lai Y.-J., Ma J.-W., Ho C.-T., Hung S.-W., Chen Y.-H., Chen C.-T., Kao J.-Y., Way T.-D. (2019). Citronellol Induces Necroptosis of Human Lung Cancer Cells *via* TNF-α Pathway and Reactive Oxygen Species Accumulation. Vivo.

[66] Garcia C., Silva C.O., Monteiro C.M., Nicolai M., Viana A., Andrade J.M., Barasoain I., Stankovic T., Quintana J., Hernández I. (2018). Anticancer Properties of the Abietane Diterpene 6,7-Dehydroroyleanone Obtained by Optimized Extraction. Future Med. Chem..

[67] Yang L., Yang N., Ran Y., Zhao H., Hu Q., Hong Y., Tian M. (2025). *Pittosporum glabratum* Leaf (Shanzhi Tea) Essential Oil: Chemical Composition, Anticancer Activities and Potential Mechanisms In Vitro and In Vivo. J. Funct. Foods.

[68] Gündoğan G.İ., Nath E.Ö. (2021). In Vitro Physiological Effects of *Origanum onites* L. (Lamiaceae) Essential Oil Treatment on Human Origin Cell Lines. Turk. J. Bot..

[69] Theeramunkong S., Utsintong M. (2018). Comparison between Volatile Oil from Fresh and Dried Fruits of *Zanthoxylum rhetsa* (Roxb.) DC. and Cytotoxicity Activity Evaluation. Pharmacogn. J..

[70] Tran V.T.K., Le T.T., Nguyen C.H., Thi Tran T.N., Hoang V. (2024). Chemical Composition, Antibacterial, Anticancer, and Anti-a-Glucosidase Activities of Essential Oils from Alpinia Nelumboides. J. Appl. Pharm. Sci..

[71] Bader A., Abdalla A.N., Obaid N.A., Youssef L., Naffadi H.M., Elzubier M.E., Almaimani R.A., Flamini G., Pieracci Y., El-Readi M.Z. (2023). In Vitro Anticancer and Antibacterial Activities of the Essential Oil of Forsskal’s Basil Growing in Extreme Environmental Conditions. Life.

[72] Guesmi F., Prasad S., Ali M.B., Ismail I.A., Landoulsi A. (2021). *Thymus hirtus* sp. Algeriensis Boiss. and Reut. Volatile Oil Enhances TRAIL/Apo2L Induced Apoptosis and Inhibits Colon Carcinogenesis through Upregulation of Death Receptor Pathway. Aging.

[73] Marković T., Popović S., Matić S., Mitrović M., Anđić M., Kočović A., Vukić M., Petrović V., Branković J., Vuković N. (2024). Insights into Molecular Mechanisms of Anticancer Activity of *Juniperus communis* Essential Oil in HeLa and HCT 116 Cells. Plants.

[74] Islam A., Chang Y.-C., Tsao N.-W., Wang S.-Y., Chueh P.J. (2024). *Calocedrus formosana* Essential Oils Induce ROS-Mediated Autophagy and Apoptosis by Targeting SIRT1 in Colon Cancer Cells. Antioxidants.

[75] Nakayama K., Murata S., Ito H., Iwasaki K., Villareal M.O., Zheng Y.-W., Matsui H., Isoda H., Ohkohchi N. (2017). Terpinen-4-Ol Inhibits Colorectal Cancer Growth via Reactive Oxygen Species. Oncol. Lett..

[76] Asif M., Yehya A.H.S., Al-Mansoub M.A., Revadigar V., Ezzat M.O., Khadeer Ahamed M.B., Oon C.E., Murugaiyah V., Abdul Majid A.S., Abdul Majid A.M.S. (2016). Anticancer Attributes of *Illicium verum* Essential Oils against Colon Cancer. S. Afr. J. Bot..

[77] Abadi A.V.M., Karimi E., Oskoueian E., Mohammad G.R.K.S., Shafaei N. (2022). Chemical Investigation and Screening of Anti-Cancer Potential of *Syzygium aromaticum* L. Bud (Clove) Essential Oil Nanoemulsion. 3 Biotech.

[78] Bernacchi A., Valerii M.C., Spigarelli R., Dussias N.K., Rizzello F., Spisni E. (2026). Investigating the Molecular Mechanisms of the Anticancer Effects of Eugenol and Cinnamaldehyde Against Colorectal Cancer (CRC) Cells In Vitro. Int. J. Mol. Sci..

[79] El-Garawani I.M., El-Nabi S.H., Dawoud G.T., Esmail S.M., Abdel Moneim A.E. (2019). Triggering of Apoptosis and Cell Cycle Arrest by Fennel and Clove Oils in Caco-2 Cells: The Role of Combination. Toxicol. Mech. Methods.

[80] Petrocelli G., Farabegoli F., Valerii M.C., Giovannini C., Sardo A., Spisni E. (2021). Molecules Present in Plant Essential Oils for Prevention and Treatment of Colorectal Cancer (CRC). Molecules.

[81] Ben Hamouda S., Zakraoui O., Souissi S., Bouzeyen R., Essafi M., Essafi-Benkhadir K. (2025). Deciphering the Mechanisms Underlying the Antitumor Effects of Eucalyptus Essential Oil and Its Component 3-Cyclohexene-1-Methanol Against Human Colon Cancer Cells. Int. J. Mol. Sci..

[82] Karousa M.M., Kalath H., Karam L., Suleman M., Ayoub M.M., Fathima A., Rocha M.A.M., Mechmechani S., Pinto D.C.G.A., Yassine H.M. (2026). *Chrysopogon zizanioides* (Vetiver) Essential Oil from Qatar Targets AKT1 and STAT3 in Colorectal and Lung Cancer: GC-MS Profiling, In Vitro Antiproliferative Activity, and In Silico Analyses. Plants.

[83] Leong W., Huang G., Liao W., Xia W., Li X., Su Z., Liu L., Wu Q., Wong V.K.W., Law B.Y.K. (2022). Traditional Patchouli Essential Oil Modulates the Host’s Immune Responses and Gut Microbiota and Exhibits Potent Anti-Cancer Effects in Apc Mice. Pharmacol. Res..

[84] Elshafie H., Armentano M., Carmosino M., Bufo S., De Feo V., Camele I. (2017). Cytotoxic Activity of *Origanum vulgare* L. on Hepatocellular Carcinoma Cell Line HepG2 and Evaluation of Its Biological Activity. Molecules.

[85] Fikry E., Orfali R., Elbaramawi S.S., Perveen S., El-Shafae A.M., El-Domiaty M.M., Tawfeek N. (2023). *Chamaecyparis lawsoniana* Leaf Essential Oil as a Potential Anticancer Agent: Experimental and Computational Studies. Plants.

[86] Khalil H.E., Ibrahim H.-I.M., Darrag H.M., Matsunami K. (2021). Insight into Analysis of Essential Oil from *Anisosciadium lanatum* Boiss.—Chemical Composition, Molecular Docking, and Mitigation of Hepg2 Cancer Cells through Apoptotic Markers. Plants.

[87] Lima E.J.S.P.D., Alves R.G., D’Elia G.M.A., Anunciação T.A.D., Silva V.R., Santos L.D.S., Soares M.B.P., Cardozo N.M.D., Costa E.V., Silva F.M.A.D. (2018). Antitumor Effect of the Essential Oil from the Leaves of *Croton matourensis* Aubl. (Euphorbiaceae). Molecules.

[88] Ali H., Al-Khalifa A.R., Aouf A., Boukhebti H., Farouk A. (2020). Effect of Nanoencapsulation on Volatile Constituents, and Antioxidant and Anticancer Activities of *Algerian origanum* Glandulosum Desf. Essential Oil. Sci. Rep..

[89] Oriola A.O. (2024). Turmeric–Black Cumin Essential Oils and Their Capacity to Attenuate Free Radicals, Protein Denaturation, and Cancer Proliferation. Molecules.

[90] Zuo H.X., Jin Y., Wang Z., Li M.Y., Zhang Z.H., Wang J.Y., Xing Y., Ri M.H., Jin C.H., Xu G.H. (2020). Curcumol Inhibits the Expression of Programmed Cell Death-Ligand 1 through Crosstalk between Hypoxia-Inducible Factor-1α and STAT3 (T705) Signaling Pathways in Hepatic Cancer. J. Ethnopharmacol..

[91] Lv D., Pan L., Zhang R., Yang J., Chen H., Wen Y., Huang M., Ma X., Wang Q., Yang X. (2020). Essential Oil from *Euphorbia esula* Inhibits Proliferation and Induces Apoptosis in HepG2 Cells via Mitochondrial Dysfunction. Braz. J. Pharm. Sci..

[92] Abd-Rabou A.A., Edris A.E. (2021). Cytotoxic, Apoptotic, and Genetic Evaluations of *Nigella sativa* Essential Oil Nanoemulsion against Human Hepatocellular Carcinoma Cell Lines. Cancer Nano.

[93] Fatima K., Luqman S., Meena A. (2022). Carvacrol Arrests the Proliferation of Hypopharyngeal Carcinoma Cells by Suppressing Ornithine Decarboxylase and Hyaluronidase Activities. Front. Nutr..

[94] Fekrazad R., Afzali M., Pasban-Aliabadi H., Esmaeili-Mahani S., Aminizadeh M., Mostafavi A. (2017). Cytotoxic Effect of *Thymus caramanicus* Jalas on Human Oral Epidermoid Carcinoma KB Cells. Braz. Dent. J..

[95] Weerapol Y., Manmuan S., Chuenbarn T., Limmatvapirat S., Tubtimsri S. (2023). Nanoemulsion-Based Orodispersible Film Formulation of Guava Leaf Oil for Inhibition of Oral Cancer Cells. Pharmaceutics.

[96] Ellithy M.M., Tarek H.E., Saleh A.M., El Azab E.F., Ibrahim M.N. (2025). Carvacrol as a Promising Topical Agent against DMBA-Induced Oral Cancer in Rats: In Vivo Study. Pak. J. Biol. Sci..

[97] Badalamenti N., Maresca V., Porrello A., Ilardi V., Postiglione A., Dentato M., De Marino E., Pollice A., Bruno M. (2025). Kenyan *Orthosiphon schimperi* Benth. Essential Oil: Chemical Composition and Cytotoxic Activity on HeLa Cells. Plants.

[98] Muñoz-Acevedo A., González M.C., De Moya Y.S., Rodríguez J.D. (2023). Volatile Metabolites of *Piper eriopodon* (Miq.) C.DC. from Northern Region of Colombia and Assessment of In Vitro Bioactivities of the Leaf Essential Oil. Molecules.

[99] Si C., Ou Y., Ma D., Hei L., Wang X., Du R., Yang H., Liao Y., Zhao J. (2022). Cytotoxic Effect of the Essential Oils from *Erigeron Canadensis* L. on Human Cervical Cancer HeLa Cells In Vitro. Chem. Biodivers..

[100] Pereira C.B., Kanunfre C.C., Farago P.V., Borsato D.M., Budel J.M., De Noronha Sales Maia B.H.L., Campesatto E.A., Sartoratto A., Miguel M.D., Miguel O.G. (2017). Cytotoxic Mechanism of *Baccharis milleflora* (Less.) DC. Essential Oil. Toxicol. Vitr..

[101] Labbozzetta M., Poma P., Occhipinti C., Sajeva M., Notarbartolo M. (2022). Antitumor Effect of *Glandora rosmarinifolia* (Boraginaceae) Essential Oil through Inhibition of the Activity of the Topo II Enzyme in Acute Myeloid Leukemia. Molecules.

[102] Bouhtit F., Najar M., Moussa Agha D., Melki R., Najimi M., Sadki K., Boukhatem N., Bron D., Meuleman N., Hamal A. (2021). New Anti-Leukemic Effect of Carvacrol and Thymol Combination through Synergistic Induction of Different Cell Death Pathways. Molecules.

[103] Momtazi A.A., Askari-Khorasgani O., Abdollahi E., Sadeghi-Aliabadi H., Mortazaeinezhad F., Sahebkar A. (2017). Phytochemical Analysis and Cytotoxicity Evaluation of *Kelussia odoratissima* Mozaff. J. Acupunct. Meridian Stud..

[104] Tian Y., Jia X., Wang Q., Lu T., Deng G., Tian M., Zhou Y. (2022). Antioxidant, Antibacterial, Enzyme Inhibitory, and Anticancer Activities and Chemical Composition of *Alpinia galanga* Flower Essential Oil. Pharmaceuticals.

[105] Costa É.R., Louro G.M., Simionatto S., Vasconcelos N.G., Cardoso C.A.L., Mallmann V., da Silva R.C.L., Matos M.F.C., Pizzuti L., Santiago E.F. (2017). Chemical Composition, Antitumoral and Antibacterial Activities of Essential Oils from Leaves and Stem Bark of *Nectandra lanceolata* (Lauraceae). J. Essent. Oil Bear. Plants.

[106] Calzada F., Ramírez-Santos J., Ordoñez-Razo R.M., Valdes M., Velázquez C., Barbosa E. (2025). Anti-Lymphoma Activity of Acyclic Terpenoids and Its Structure–Activity Relationship: In Vivo, In Vitro, and In Silico Studies. Int. J. Mol. Sci..

[107] Thalappil M.A., Butturini E., Carcereri De Prati A., Bettin I., Antonini L., Sapienza F.U., Garzoli S., Ragno R., Mariotto S. (2022). Pinus Mugo Essential Oil Impairs STAT3 Activation through Oxidative Stress and Induces Apoptosis in Prostate Cancer Cells. Molecules.

[108] Ray A., Gadnayak A., Jena S., Sahoo A., Patnaik J., Chandra Panda P., Nayak S. (2023). Hedychium Spicatum Rhizome Essential Oil Induces Apoptosis in Human Prostate Adenocarcinoma PC-3 Cells via Mitochondrial Stress and Caspase Activation. Heliyon.

[109] Arul S., Rajagopalan H., Ravi J., Dayalan H. (2020). Beta-Caryophyllene Suppresses Ovarian Cancer Proliferation by Inducing Cell Cycle Arrest and Apoptosis. Anti-Cancer Agents Med. Chem..

[110] Brunetti A., Marinelli O., Morelli M.B., Iannarelli R., Amantini C., Russotti D., Santoni G., Maggi F., Nabissi M. (2019). Isofuranodiene Synergizes with Temozolomide in Inducing Glioma Cells Death. Phytomedicine.

[111] Spigarelli R., Spisni E., Magalhães M., Cabral C., Gonçalves A.C., Saracino I.M., Botti G., Dalpiaz A., Beggiato S., Valerii M.C. (2024). Clove Essential Oil as a Source of Antitumoral Compounds Capable of Crossing the Blood–Brain Barrier: A Focus on the Effects of β-Caryophyllene and Eugenol in a Glioblastoma Cell Line. Int. J. Mol. Sci..

[112] Pincigher L., Valenti F., Bergamini C., Prata C., Fato R., Amorati R., Jin Z., Farruggia G., Fiorentini D., Calonghi N. (2023). Myrcene: A Natural Compound Showing Anticancer Activity in HeLa Cells. Molecules.

[113] Catalani S., Palma F., Battistelli S., Benedetti S. (2017). Oxidative Stress and Apoptosis Induction in Human Thyroid Carcinoma Cells Exposed to the Essential Oil from *Pistacia lentiscus* Aerial Parts. PLoS ONE.

[114] Hosseini K., Jasori S., Delazar A., Asgharian P., Tarhriz V. (2021). Phytochemical Analysis and Anticancer Activity of Falcaria Vulgaris Bernh Growing in Moghan Plain, Northwest of Iran. BMC Complement. Med. Ther..

[115] Xavier A.L., Pita J.C.L.R., Brito M.T., Meireles D.R.P., Tavares J.F., Silva M.S., Maia J.G.S., Andrade E.H.A., Diniz M.F.F.M., Silva T.G. (2015). Chemical Composition, Antitumor Activity, and Toxicity of Essential Oil from the Leaves of *Lippia microphylla*. Z. Naturforschung C.

[116] MasodKhooy M.J., Farasat M., Salehi Salmi M., Mirzaei H. (2023). Combinatorial Treatment with *Silybum marianum* Essential Oil Enhances the Therapeutic Efficacy of a 5-fluorouracil Base Therapy for Hepatocellular Carcinoma. Phytother. Res..

[117] Dangkong D., Limpanasithikul W. (2015). Effect of Citral on the Cytotoxicity of Doxorubicin in Human B-Lymphoma Cells. Pharm. Biol..

[118] Zeng C., Fan D., Xu Y., Li X., Yuan J., Yang Q., Zhou X., Lu J., Zhang C., Han J. (2020). Curcumol Enhances the Sensitivity of Doxorubicin in Triple-Negative Breast Cancer via Regulating the miR-181b-2-3p-ABCC3 Axis. Biochem. Pharmacol..

[119] Younis N.S., Elsewedy H.S., Soliman W.E., Shehata T.M., Mohamed M.E. (2021). Geraniol Isolated from Lemon Grass to Mitigate Doxorubicin-Induced Cardiotoxicity through Nrf2 and NF-κB Signaling. Chem.-Biol. Interact..

[120] Edris A.E. (2007). Pharmaceutical and Therapeutic Potentials of Essential Oils and Their Individual Volatile Constituents: A Review. Phytother. Res..

[121] Sitarek P., Rijo P., Garcia C., Skała E., Kalemba D., Białas A.J., Szemraj J., Pytel D., Toma M., Wysokińska H. (2017). Antibacterial, Anti-Inflammatory, Antioxidant, and Antiproliferative Properties of Essential Oils from Hairy and Normal Roots of *Leonurus sibiricus* L. and Their Chemical Composition. Oxidative Med. Cell. Longev..

[122] Tit D.M., Bungau S.G. (2023). Antioxidant Activity of Essential Oils. Antioxidants.

[123] Blowman K., Magalhães M., Lemos M.F.L., Cabral C., Pires I.M. (2018). Anticancer Properties of Essential Oils and Other Natural Products. Evid.-Based Complement. Altern. Med..

[124] Panda S., Sahoo S., Tripathy K., Singh Y.D., Sarma M.K., Babu P.J., Singh M.C. (2022). Essential Oils and Their Pharmacotherapeutics Applications in Human Diseases. Adv. Tradit. Med..

[125] Guzmán E., Lucia A. (2021). Essential Oils and Their Individual Components in Cosmetic Products. Cosmetics.

[126] Bunse M., Daniels R., Gründemann C., Heilmann J., Kammerer D.R., Keusgen M., Lindequist U., Melzig M.F., Morlock G.E., Schulz H. (2022). Essential Oils as Multicomponent Mixtures and Their Potential for Human Health and Well-Being. Front. Pharmacol..

[127] Ramsey J.T., Shropshire B.C., Nagy T.R., Chambers K.D., Li Y., Korach K.S. (2020). Essential Oils and Health. Yale J. Biol. Med..

[128] Blagosklonny M.V. (2023). Selective Protection of Normal Cells from Chemotherapy, While Killing Drug-Resistant Cancer Cells. Oncotarget.

[129] Chen X. (2000). Protection of Normal Proliferating Cells Against Chemotherapy by Staurosporine-Mediated, Selective, and Reversible G1 Arrest. J. Natl. Cancer Inst..

[130] Cheok C.F. (2012). Protecting Normal Cells from the Cytotoxicity of Chemotherapy. Cell Cycle.

[131] Hengstler J.G., Van Der Burg B., Steinberg P., Oesch F. (1999). Interspecies differences in cancer susceptibility and toxicity*. Drug Metab. Rev..

[132] Laskin J.D., Evans R.M., Slocum H.K., Burke D., Hakala M.T. (1979). Basis for Natural Variation in Sensitivity to 5-Fluorouracil in Mouse and Human Cells in Culture1. Cancer Res..

[133] Lee T.C., Ho I.C. (1994). Differential Cytotoxic Effects of Arsenic on Human and Animal Cells. Environ. Health Perspect..

[134] Haglund C., Åleskog A., Nygren P., Gullbo J., Höglund M., Wickström M., Larsson R., Lindhagen E. (2012). In Vitro Evaluation of Clinical Activity and Toxicity of Anticancer Drugs Using Tumor Cells from Patients and Cells Representing Normal Tissues. Cancer Chemother. Pharmacol..

[135] Jacus M.O., Daryani V.M., Harstead K.E., Patel Y.T., Throm S.L., Stewart C.F. (2016). Pharmacokinetic Properties of Anticancer Agents for the Treatment of Central Nervous System Tumors: Update of the Literature. Clin. Pharmacokinet..

